# Study on chemical constituents and antioxidant activities of *Dianthus caryophyllus* L.

**DOI:** 10.3389/fpls.2024.1438967

**Published:** 2024-08-22

**Authors:** Miaomiao Wang, Qiuyu Shen, Jianyu Pang, Yu Mao, Xiaofei Li, Yanxia Tao, Wenru Tang, Ruifen Sun, Xuhong Zhou

**Affiliations:** ^1^ Office of Science and Technology, Yunnan University of Chinese Medicine, Kunming, Yunnan, China; ^2^ Department of Pharmacy, Bozhou Hospital of Traditional Chinese Medicine, Bozhou, Anhui, China; ^3^ Laboratory of Molecular Genetics of Aging and Tumor, Medical Faculty, Kunming University of Science and Technology, Kunming, Yunnan, China

**Keywords:** carnation, flavonoids, antioxidant activity, metabolomics, molecular docking

## Abstract

**Objective:**

*Carnation* is a plant that holds high value in terms of its edibility, medicinal properties, and ornamental appeal. Creating no sense he aim of this study was to evaluate the antioxidant and antitumor properties of extracts derived from various parts of the carnation plant. Metabolomics technology was employed to identify the primary chemical constituents.

**Methods:**

Initially, we measured the total phenolic and total flavonoid contents in carnation roots, stems, leaves, and flowers, followed by assessing the antioxidant and anti-tumor capabilities of each component using diverse experimental methods. Subsequently, UPLC-MS/MS was employed to identify metabolites in different parts of carnation and investigate their roles in antioxidant and anti-tumor activities.

**Results:**

Mention numerical value- for better underatnding- Results of the study indicated that the methanol extract obtained from carnation flowers and roots exhibited superior antioxidant capacity compared to that from the stems and leaves. This disparity may be attributed to the abundance of polyphenols, flavonoids, and antioxidants present in the flowers, including methyl ferulate and luteolin-4’-O-glucoside. Furthermore, the significant presence of the anthraquinone compound rhein-8-O-glucoside in carnation roots may contribute to their enhanced antioxidant properties. Ten distinct compounds were isolated and recognized in carnation flowers, with Isoorientin 2”-O-rhamnoside and Kurarinone demonstrating notable antioxidant activity and binding affinity to SOD1 and SOD3, as validated through antioxidant screening and molecular docking.

**Conclusion:**

Overall, the findings from this study have expanded our knowledge of the phytochemical composition across different anatomical regions of the carnation plant, providing valuable insights for its holistic utilization.

## 1 Introduction

*Dianthus caryophyllus L*., a perennial herb of the Dianthus genus in the Caryophyllaceae family, is commonly prized for its use as a cut flower (Chrysargyris et al., 2024). Beyond its ornamental appeal, carnations hold significant value in culinary and medicinal domains. They are brewed into teas, wines, porridges, and other culinary delights, imparting both a fragrant flavor and aesthetic appeal. *Dianthus caryophyllus L* is a winter annual plant with a short to medium height. The plant grows upright with thin dark green leaves on the stem (Al-Zurfi et al., 2023). In traditional medicine, both domestically and internationally, carnations have been utilized to treat various ailments such as sore throats, gum infections, aid in wound healing, manage gastrointestinal disorders, and address numerous other health issues (Mohammed and Al-Bayati, 2009; Martineti et al., 2010; Chandra et al., 2016). Many diseases arise from an excess of free radicals in the body, which damage normal cells and tissues, contributing to conditions like heart disease, cancer, Parkinson’s disease, and Alzheimer’s disease. Antioxidants are pivotal in neutralizing free radical damage within the human body, thereby slowing the aging process and reducing cancer risk.

Currently, research on carnations primarily focuses on aboveground extracts and specific volatile components, with little differentiation among different tissue parts. The underlying material basis for the pharmacological effects of carnation extracts remains unclear, thus limiting their further development and utilization.

Metabolomics, distinguished for its high sensitivity, selectivity, and precision, is widely applied in clinical disease diagnostics, drug safety evaluation, botanical studies, nutrition, and numerous other fields (Sébédio, 2017; Wang et al., 2017; Szűcs et al., 2019; Karkoula et al., 2020). Various analytical techniques such as GC-MS, LC-MS, and NMR are employed for species differentiation and metabolic analysis of different plant parts (Pace et al., 2015). In traditional Chinese medicine, metabolomics has emerged as a rapid and high-throughput method to understand bioactive metabolites (Zampieri et al., 2017). The systematic and comprehensive metabolomic assessment of medicinal plants significantly supports the ongoing enhancement of their medicinal value.

The objective of this study was to assess the antioxidant and antitumor properties of extracts from various parts of the carnation plant. Metabolomics technology was utilized to identify the primary chemical constituents present in the roots, stems, leaves, and flowers, aiming to uncover the pharmacological basis of these extracts.

In addition, a range of chromatographic techniques—including column chromatography on silica gel and glucan gel, as well as high-performance liquid chromatography—was employed for the isolation and purification of active compounds from carnation. These methods were complemented by advanced spectral analysis techniques such as ultraviolet spectroscopy, nuclear magnetic resonance spectroscopy, and mass spectrometry for compound structure determination. The identified compounds were screened to evaluate their antioxidant properties. Molecular docking studies were also conducted to explore the potential antioxidants, using oxidation-related gene sets to validate their efficacy. These efforts lay a scientific foundation for future research, development, and expansion of carnation resources.

## 2 Experimental procedure

### 2.1 Chemicals and reagents

2,2-Diphenyl-1-pyridine hydrazine radical (DPPH) was procured from Phygene Life Sciences Co., Ltd. The Folin-Ciocalteau reagent, 2,2’-azinobis(3-ethylbenzothiazoline-6-sulfonic acid) diammonium salt (ABTS), and 2,4,6-tri(2-pyridyl)-1,3,5-triazine (TPTZ) were obtained from Macklin (Shanghai, China). Rutin and gallic acid were sourced from Solarbio (Beijing, China). Analytical grade methanol, glacial acetic acid, and ferric chloride (FeCl3·6H2O) were purchased from Tianjin Zhiyuan Chemical Reagent Co., Ltd. Potassium persulfate was acquired from United Initiator (Shanghai) Co., Ltd. Sodium acetate trihydrate was obtained from Aladdin (Shanghai, China). L-Ascorbic acid (VC) was sourced from Guangdong Guanghua Technology Co., Ltd. Chromatographic grades of methanol, acetonitrile, and RP-C18 40~63 μm were supplied by Merck (Germany). RPMI-1640, trypsin, PBS buffer, and penicillin-streptomycin solution were purchased from BI Shanghai Co., Ltd. Fetal bovine serum (FBS) was obtained from Cyagen Biosciences Inc (America). DMSO was acquired from Solarbio (Beijing, China). The CCK-8 assay kit was purchased from Proteintech Group, Inc., while the Annexin V-FITC/PI dual-staining fluorescence apoptosis detection kit was obtained from Procell Life Science & Technology Co., Ltd. (Wuhan, China). Cisplatin (DDP) was sourced from Shanghai Yuanye Bio-Technology Co., Ltd.

Chromatographic grade standards for metabolite identification were obtained from BioBioPha (Kunming, China) and Sigma-Aldrich (St. Louis, United States). Silica gel for column chromatography (80–100 mesh and 200–300 mesh) was purchased from Qingdao Kangyexin Medicated Silica Gel Desiccant Co., Ltd. GF254 silicone plates measuring 50mm x 100mm were procured from Linyi Haixiang Chemical Co., Ltd. The macroporous adsorbent resin was acquired from Xi’an Lanxiao Technology New Material Co., Ltd. Sephadex LH-20 was sourced from Amersham Biosciences (Sweden). Additionally, a preparative reverse-phase column measuring 250 mm x 20 mm with a particle size of 10 μm was obtained from Daisogel (Japan).

### 2.2 Sample source and preparation

#### 2.2.1 Preparation of samples for biological activity assay and UPLC-MS/MS analysis

Fresh roots, stems, leaves, and flowers of *Dianthus caryophyllus L*. (R-Dc, S-Dc, L-Dc, and F-Dc) were harvested in Kunming, Yunnan Province, China, and subsequently subjected to freeze-drying (**Supplementary Figure 1**). Once completely dried, they were processed into a powder form (30 Hz, 1.5 min). A suitable quantity of powder from each sample was then weighed and dissolved in a 70% methanol solution (solid-liquid ratio 1:12), followed by vortexing for 30 seconds at 30-minute intervals for six cycles overnight (4°C). For antioxidant and anticancer assays, the samples underwent filtration, spin-drying, and were stored at 4°C until required. Samples designated for UPLC-MS/MS analysis were centrifuged (12000 rpm, 10 min), filtered (0.22 μm), and the resulting supernatant was collected and stored in sample vials for UPLC-MS/MS analysis.

#### 2.2.2 Compound separation sample preparation

Fresh carnation flowers weighing 20 kg were cold-soaked in twice the volume of 95% ethanol for extraction. The first extraction lasted 7 days, followed by a 5-day second extraction, and a 2-day third extraction. The filtrates from all extractions were combined and subjected to rotary evaporation to yield a 95% ethanol extract, which was then utilized for compound separation and purification.

### 2.3 Total phenolic and flavonoid content

The total phenolic content (TPC) of extracts from R-Dc, S-Dc, L-Dc, and F-Dc was determined using the Folin-Ciocalteau method. Specifically, 0.5 mL of each extract was mixed with 0.5 mL of Folin–Ciocalteau reagent in a 10 mL centrifuge tube, followed by the addition of 1 mL of 7.5% Na2CO3 solution. The resulting solution was diluted with distilled water to a total volume of 10 mL. The reaction mixture was then left to incubate in the dark for 2 hours (Dulf et al., 2015), and subsequently centrifuged at 8000 rpm at 4°C for 10 minutes. The supernatant was collected and the absorbance was measured at 765 nm (Xie et al., 2021). Gallic acid served as the standard, and the results were expressed as milligrams of gallic acid per gram of sample (mg/g).

The total flavonoid content (TFC) in the samples was quantified using the AlCl3 method. Initially, 0.5 mL of the extract solution was placed in a 10 mL centrifuge tube, and 0.5 mL of 5% NaNO2 solution was added. The mixture incubated in darkness for 5 minutes, followed by the addition of 0.5 mL 10% AlCl3. After an additional 1-minute incubation in the dark, 5 mL of 1 mol/L NaOH was promptly added, and distilled water was used to adjust the solution volume to 10 mL. The reaction proceeded in darkness at room temperature for 15 minutes, and then the solution underwent centrifugation at 8000 rpm at 4°C for 10 minutes. The absorbance value was measured at 510 nm by collecting the supernatant (Dulf et al., 2016). The flavonoid content, reported as mg of rutin per gram of the sample (mg/g), was determined using rutin as a reference standard.

### 2.4 Antioxidant assays

#### 2.4.1 DPPH radical scavenging assay

Referring to the DPPH free radical scavenging methods described in the literature and implementing necessary enhancements (Zhang et al., 2022), DPPH solution was utilized as the substrate at a concentration of 0.15 mmol/L. The solution was then allocated into three distinct groups: Group A, the negative control (comprising of 160 μL methanol and 40 μL DPPH solution); Group B, the sample group (containing either 160 μL sample or a positive control - VC - and 40 μL DPPH solution); and Group C, the sample control (consisting of 160 μL sample and 40 μL methanol). These components were sequentially dispensed into a 96-well plate. Following a 30-minute incubation period for a complete reaction, the absorbance was measured at 517 nm. The DPPH free radical scavenging activity was quantified using the formula: DPPH clearance rate = [(A - (B - C))/A] × 100%. The IC50 value was calculated using SPSS software.

#### 2.4.2 ABTS^+^ radical scavenging assay

After combining equal volumes of 7 mM ABTS^+^ solution and 2.45 mM potassium persulfate solution, the mixture was stored at 4°C in the dark for 12–16 hours. Subsequently, it was diluted with methanol until the absorbance, measured at 734 nm using a microplate reader, reached 0.7 ± 0.05. Each extract was diluted to various concentrations for different groups: the Blank group (consisting of 100 μL methanol and 100 μL ABTS^+^ solution), the experimental group (comprising 100 μL sample or VC and 100 μL ABTS^+^ solution), and the experimental control group (with 100 μL sample and 100 μL methanol) were sequentially added to 96-well plates. After incubating the reaction in darkness for 30 minutes, the absorbance was recorded at 734 nm. Three parallel experiments were conducted (Huang et al., 2005). The ABTS^+^ radical scavenging activity was determined using the same calculation method as outlined in the DPPH assay.

#### 2.4.3 Determination of iron reducing power (FRAP)

The standard FeSO_4_ stock solution with a concentration of 4 mmol/L was diluted to concentrations of 0.0125, 0.025, 0.05, 0.1, 0.2, 0.4, 0.6, 0.8, and 1.2 mmol/L using methanol as the solvent. To construct a standard curve, the absorbance at 593 nm was recorded, with FeSO_4_ concentration on the horizontal axis and the absorbance of (Ai-Aj) on the vertical axis.

The FRAP working solution was prepared, and extracts from distinct parts of the carnation were utilized at various concentrations. The samples were divided into two groups: Group Ai (containing 150 μL FRAP and 50 μL of the sample) and Group Aj (containing 150 μL FRAP and 50 μL methanol), which were then added to the 96-well plates consecutively. Subsequently, the samples were allowed to fully react at 37°C for 10 minutes, followed by the determination of their absorption value at 593 nm (Biskup et al., 2013). Vitamin C (VC) served as the positive control, with three replicate samples for each instance.

In determining the total antioxidant capacity, the absorbance measurements of the sample (Ai) and the blank control group (Aj) are utilized in the FeSO4 standard curve equation. Ai is the absorbance of the sample to be measured and Aj was the absorbance of the blank control group.

#### 2.4.4 Statistical analysis

The analysis and processing of data in this study were conducted utilizing SPSS and GraphPad Prism software. The results were presented as mean ± standard deviation ( $\bar{x}$ ± *s*). The relationship between polyphenol and flavonoid content and antioxidant capacity was evaluated using the Spearman correlation coefficient.

### 2.5 Anticancer experiment

#### 2.5.1 CCK-8 assay for detecting anticancer activity

The human non-small cell lung cancer cell line A549 (obtained from Procell Life Science & Technology Co., Ltd.) and the human osteosarcoma cell line U2OS (obtained from the Kunming Cell Bank, Chinese Academy of Sciences) were cultured in RPMI-1640 medium supplemented with 10% fetal bovine serum (FBS) and 1% penicillin-streptomycin solution. The cells were maintained in a 5% CO_2_ humidified incubator at 37°C. A549 and U2OS cells were seeded at a density of 10,000 cells per well in 96-well plates. The cells were treated with various concentrations of R-Dc, S-Dc, L-Dc, and F-Dc extract solutions for a specific duration. Cell viability was assessed using the CCK-8 assay.

#### 2.5.2 Cell apoptosis assay

A549 cells were exposed to varying concentrations of R-Dc and F-Dc (0 μg/mL, 30 μg/mL, and 60 μg/mL), while U2OS cells were treated with R-Dc and F-Dc at concentrations of 0 μg/mL, 15 μg/mL, and 30 μg/mL. After 24 hours, the cells were harvested for analysis. Cell apoptosis was evaluated using propidium iodide (PI) and Annexin V-fluorescein isothiocyanate (FITC) apoptosis detection reagents, and flow cytometry was utilized to assess the apoptosis levels.

#### 2.5.3 Statistical analysis

The experimental data were analyzed with SPSS and GraphPad Prism software for cell viability analysis. The results were reported as mean ± standard deviation (M ± SD). FlowJo software was employed for analyzing the flow cytometry data, with statistical significance set at P < 0.05.

### 2.6 UPLC-MS/MS analysis

#### 2.6.1 UPLC conditions

The sample extracts were analyzed using an UPLC-ESI-MS/MS system (UPLC, SHIMADZU Nexera X2, www.shimadzu.com.cn/; MS, Applied Biosystems 4500 Q TRAP, www.appliedbiosystems.com.cn/). UPLC analysis conditions are shown in the references (Zhou et al., 2023). The effluent was alternatively connected to an ESI-triple quadrupole-linear ion trap (QTRAP)-MS (Qian et al., 2023).

#### 2.6.2 ESI-Q TRAP-MS/MS

LIT and triple quadrupole (QQQ) scans were obtained using a triple quadrupole-linear ion trap mass spectrometer (Q TRAP), specifically the AB4500 Q TRAP UPLC/MS/MS System. This instrument was outfitted with an ESI Turbo Ion-Spray interface and operated in both positive and negative ion modes. Data acquisition was facilitated through Analyst 1.6.3 software developed by AB Sciex. The ESI source parameters were established as per the manufacturer’s recommendations (Li et al., 2021; Segla Koffi Dossou et al., 2022).

### 2.7 Metabonomics analysis

#### 2.7.1 Qualitative and quantitative analysis of metabolites

Based on the self-built MWDB (metware database) and MRM, the qualitative and quantitative analysis of the metabolites was carried out by mass spectrometry.

#### 2.7.2 Principal component analysis

Unsupervised Principal Component Analysis (PCA) is processed using the built-in statistical prcomp function of R software (www.r-project.org/).

#### 2.7.3 Hierarchical cluster analysis and Pearson correlation coefficient

The results from the Hierarchical Cluster Analysis (HCA) of samples and metabolites are illustrated using either a heat map or tree graph. In addition, the Pearson correlation coefficient among samples is calculated using the cor function in R and visualized exclusively as a heat map. Both the HCA and Pearson correlation coefficients are visualized with the assistance of the Pheatmap function from the R package.

#### 2.7.4 Select differential metabolites

Orthogonal Partial Least Squares-Discriminant Analysis (OPLS-DA) was utilized following the PCA outcomes to discern metabolite variances among groups and within group samples (Chen et al., 2009). The original data were log2 converted before OPLS-DA underwent centralized processing. Subsequently, the OPLS-DA model was analyzed using the OPLSR package in Metabo Analyst within the R software environment. The metabolic data were assessed in accordance with the OPLS-DA model, and corresponding score charts for each group were generated, further illustrating the differences between the groups (Thévenot et al., 2015). The Variable Importance in Projection (VIP) values of the OPLS-DA model, derived through multivariate analysis, enabled the preliminary identification of differential metabolites among distinct tissues. Concurrently, the integration of p-values or Fold Change (FC) data from univariate analysis facilitated the refinement of key differential metabolites. K-means cluster analysis was applied to categorize these key metabolites, while the Spearman correlation coefficient was utilized to examine the relationship between significant differentially expressed metabolites and their associated functional activities.

#### 2.7.5 KEGG annotation and enrichment analysis

Through KEGG database (http://www.kegg.jp/kegg/compound/) for identification of metabolites, and map the annotation of metabolites to KEGG Pathway databases (http://www.kegg.jp/kegg/pathway.html). Subsequently, the significantly regulated pathways were analyzed using Metabolite Set Enrichment Analysis (MSEA), where the significance was established based on the P-value from hypergeometric tests.

### 2.8 Separation and purification of compounds

The 95% ethanol extract of carnation underwent a series of purification steps including silica gel column chromatography, macroporous adsorption resin, Sephadex LH-20 column chromatography, semi-preparative high-performance liquid chromatography, among others (**Supplementary Figure 2**). The compounds were characterized using techniques such as NMR and MS.

### 2.9 Molecular docking

Firstly, the compound information was obtained from the PubChem database, and the 3D structure of the compound was downloaded based on the most suitable match. Next, gene sets related to antioxidant pathways were sourced from the KEGG Pathway and Gene Ontology (GO) databases. Specific antioxidant genes such as SOD, CAT, GPX, and GCLC were identified in the NCBI Gene database along with relevant literature on genes and antioxidants from PubMed. Protein structures corresponding to these gene sets were then retrieved from the UniProt database. Ultimately, the findings were visualized using the open-source version of PyMol.

## 3 Results

### 3.1 Total phenolic and flavonoid content

**Figure 1:**
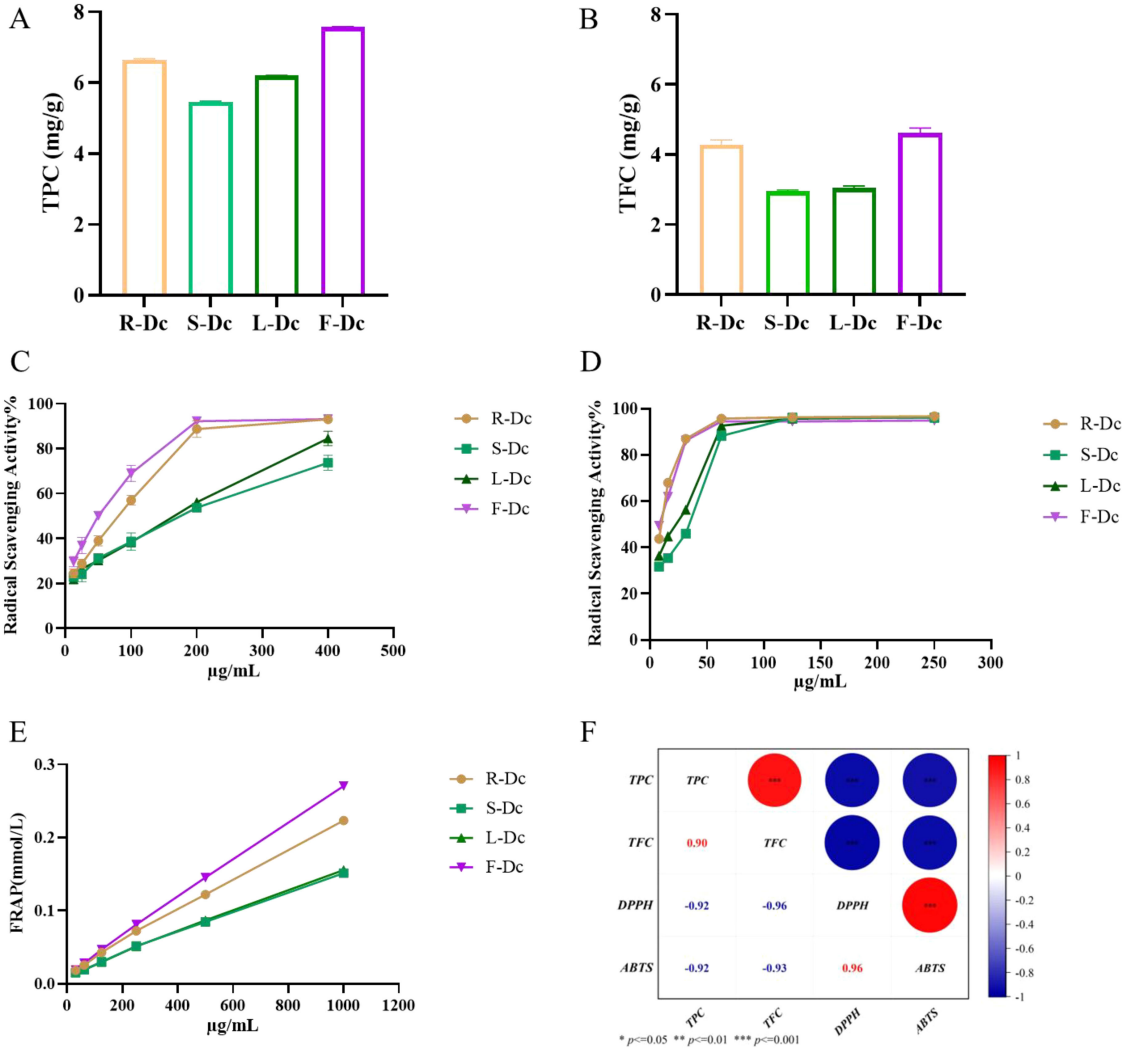
The following parameters were measured to determine the phenolic content, antioxidant capacity, and its correlation in different parts of the carnation: **(A)** Total phenolic content (TPC); **(B)** Total flavonoid content (TFC); **(C)** DPPH free radical scavenging rate; **(D)** ABTS free radical scavenging rate; **(E)** Ferric ion reducing antioxidant power (FRAP); **(F)** A heat map visualizing the correlation between TPC/TFC and antioxidant capacity.

Total polyphenol content in R-Dc, S-Dc, L-Dc, and F-Dc was illustrated in **Figure 1A**. The TPC of each sample was 5.466 ± 0.018 mg/g ~ 7.573 ± 0.016 mg/g, of which F-Dc had the highest TPC. The total flavonoid content of R-Dc, S-Dc, L-Dc and F-Dc is shown in **Figure 1B**. The TFC of each sample was 2.952 ± 0.037 mg/g ~ 4.616 ± 0.141mg/g, of which F-Dc has the highest TFC. The same trend was observed between TPC and TFC in different tissue parts of the carnation, F-Dc >R-Dc > L-Dc >S-Dc.

### 3.2 Antioxidant abilities

DPPH, ABTS free radical scavenging capacity, and FRAP were used to synthesize and assess the antioxidant activity of the different parts of the carnation. We found that roots, stems, leaves, and flowers (R-Dc, S-Dc, L-Dc and F-Dc) of the carnation had differing degrees of antioxidant capacity (**Figures 1C, D**). Among them, the scavenging effect of ABTS free radical in different parts was stronger than that of DPPH free radical. Through DPPH and ABTS free radical scavenging experiments, it could be concluded that the rank order of antioxidant capacity was as follows: F-Dc >R-Dc > L-Dc >S-Dc (**Supplementary Table 1**). Notably, the reducing capacity of iron ions in different parts of the carnation showed the same trend as the scavenging capacity of the free radicals (**Figure 1E**). Of the three antioxidant methods, all experimental results showed F-Dc to have the highest antioxidant capacity.

To clarify the relationship between TPC, TFC, and antioxidant capacity, we performed a further correlation analysis, and the results can be seen in **Figure 1F**. IC_50_ reflects the ability of DPPH and ABTS to scavenge free radicals, and the lower the IC_50_ value, the stronger the antioxidant capacity. Based on the correlation coefficient, TPC/TFC was highly correlated with different antioxidant capacities. These findings suggest that phenols and flavonoids play a crucial role in determining antioxidant capacity, which accounts for the consistent trend observed between TPC/TFC and antioxidant capacity.

### 3.3 Antitumor activity

**Figure 2:**
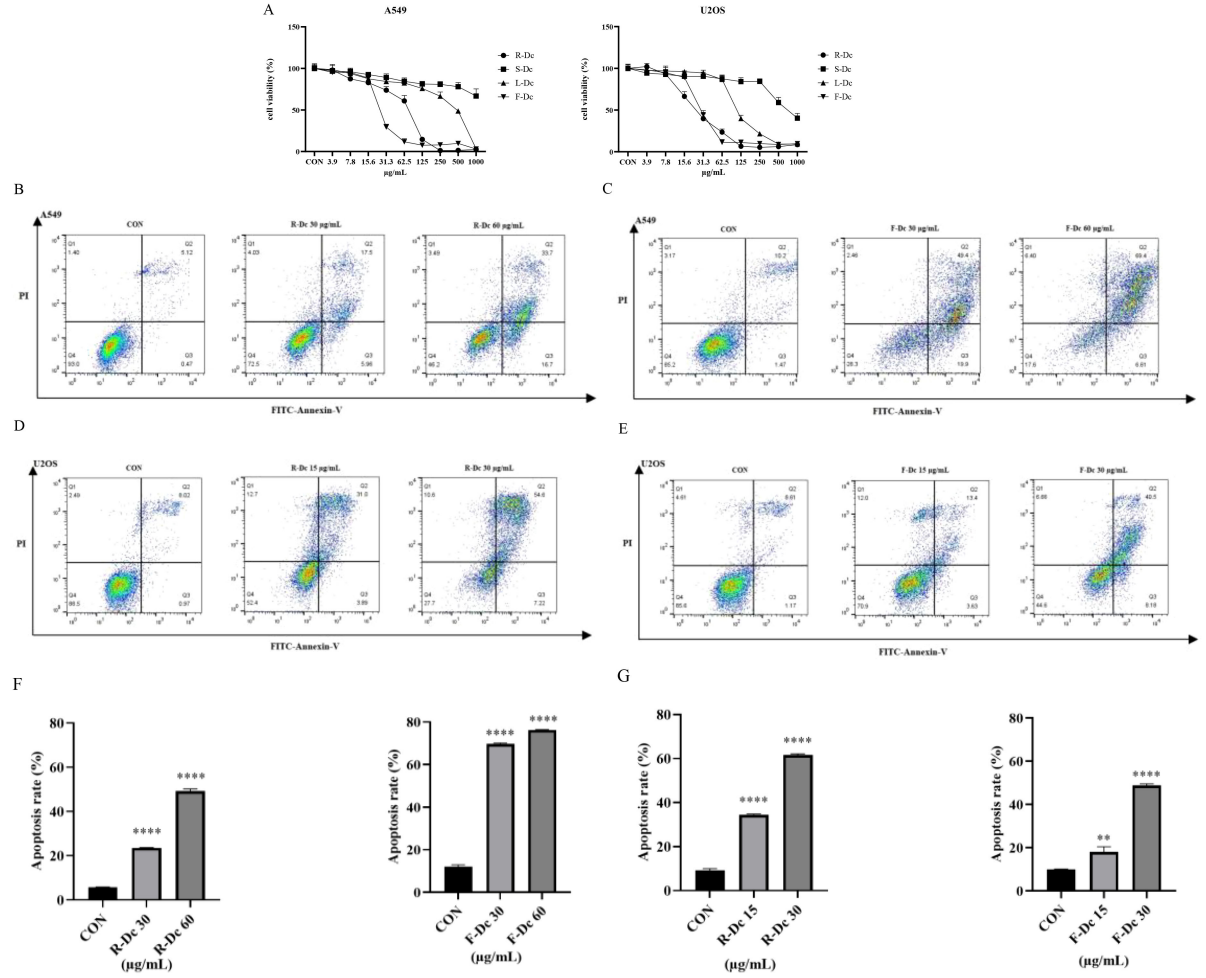
Antitumor activity of extracts from different parts of carnation: **(A)** Effects of different parts of carnation on cell viability of A549 and U2OS; **(B)** Flow cytometry of A549 cells affected by different concentrations of R-Dc; **(C)** Flow cytometry of A549 cells affected by different concentrations of F-Dc; **(D)** Flow cytometry of U2OS cells affected by different concentrations of R-Dc; **(E)** Flow cytometry of U2OS cells affected by different concentrations of R-Dc; **(F)** Statistical analysis of A549 cells treated with R-Dc and F-Dc at different concentrations for 24h; **(G)** Statistical analysis of U2OS cells treated with different concentrations of R-Dc and F-Dc for 24h. Compared with the blank group, there was significant difference. ^**^ *P*<0.01,^****^ *P*<0.0001.

The CCK-8 assay results (**Figure 2A**) showed that compared to the control group, the extracts of different parts of carnation were able to inhibit the proliferation of A549 and U2OS tumor cells. However, U2OS cell line was more sensitive, and the inhibitory effect became stronger with the increase of drug concentration. After 24h of drug intervention, the semi-inhibitory concentration (IC_50_) value of A549 cells was in the order of F-Dc < R-Dc < L-Dc < S-Dc, and the IC_50_ value of U2OS cells was in the order of R-Dc < F-Dc < L-Dc < S-Dc (**Supplementary Table 2**).

The results of flow cytometry analysis on the apoptosis of A549 cells treated with R-Dc and F-Dc extracts of carnation are shown in (**Figures 2B, C**). As the drug concentration increased, both the low-dose group (30 μg/mL) and the high-dose group (60 μg/mL) showed a significant increase in apoptosis rate and exhibited significant differences (*P* < 0.05) (**Supplementary Table 3**).

The results of apoptosis in U2OS cells treated with different concentrations of R-Dc and F-Dc after 24 hours are shown in (**Figures 2D, E**). The low-dose group (A549: 30 μg/mL and U2OS:15 μg/mL) and high-dose group (A549: 60 μg/mL and U2OS:30 μg/mL) showed a significant increase in the number of cells in the Q2 and Q3 regions compared to the control group (0 μg/mL) (**Supplementary Table 3**, **Figures 2F, G**), and the difference was statistically significant. Therefore, the extracts of R-Dc and F-Dc from carnation can significantly induce apoptosis in tumor cells A549 and U2OS.

### 3.4 Metabolic profiling of *Dianthus Caryophyllus* L. based on UPLC-MS/MS

To investigate the chemical composition in roots, stems, leaves and flowers of *Dianthus Caryophyllus* L., both primary and secondary metabolites were identified using UPLC-MS/MS analysis (**Supplementary Figure 3**). Quantitative metabolite analysis was performed using the Analyst 1.6.3 software package under multiple reaction monitoring modes **Supplementary Figure 4**.

**Figure 3:**
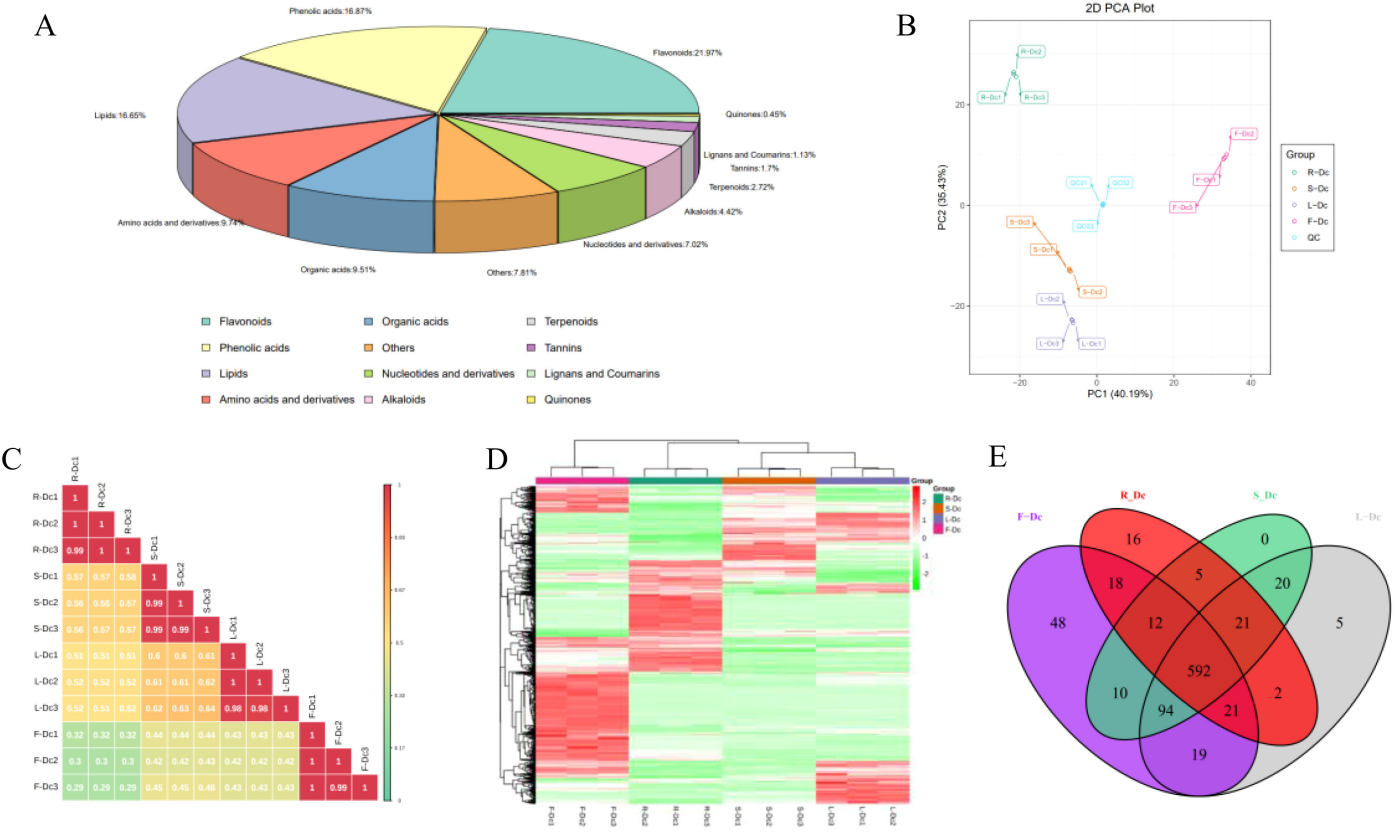
Analysis of metabolites in different tissues of carnation: **(A)** Overview of the identified metabolites in *Dianthus Caryophyllus* L.; **(B)** PCA score chart of quality spectrum data of each group of samples and quality control samples; **(C)** Plot of correlation between samples; **(D)** Population cluster of metabolites; **(E)** Venn plots of metabolites from different parts of the carnation.

A total of 883 compounds were preliminarily identified from the four parts of *Dianthus Caryophyllus* L. (**Supplementary Table 4**), including 194 flavonoids, 149 phenolic acids, 147 lipids, 86 amino acids and derivatives, 84 organic acids, 62 nucleotides and derivatives, 39 alkaloids, 24 terpenoids, 15 tannins, 10 lignans and coumarins, 4 quinones and 69 others. Among them, flavonoids (21.97%), phenolic acids (16.87%) and lipids (16.65%) were the three main metabolites. The flavonoids and lipids could be further categorized into ten and six classes, respectively (**Figure 3A**; **Supplementary Table 5**).

### 3.5 Multivariate statistical analysis of the metabolites of carnation

Through PCA analysis of samples (including QC samples), we gained preliminary insights into the overall metabolic differences between sample groups and the degree of variation within each group. It is noteworthy that the closely clustered blue QC points indicate system stability. PCA results demonstrated distinct clustering of R-Dc, S-Dc, L-Dc, and F-Dc samples, indicating significant metabolic differences among different tissue parts of carnation (**Figure 3B**). Pearson correlation analysis revealed significant correlations between biological replicates of R-Dc, S-Dc, L-Dc, and F-Dc (**Figure 3C**). The heatmap of metabolite clustering clearly illustrated similarities between biological replicates and differences across different tissue parts. Hierarchical clustering analysis further delineated R-Dc, S-Dc, L-Dc, and F-Dc into distinct groups, highlighting differences in metabolic accumulation patterns among these groups (**Figure 3D**).

To get insight into the metabolic differences and identify differential metabolites between each group, we performed an OPLS-DA analysis. The score plots of the pairwise comparisons are presented in **Supplementary Figure 5**. With the exception of the between-group comparison, we found high predictability (Q2) and high goodness of fit (R2X, R2Y). For instance, the Q2 values between R-DC and S-DC, R-DC and L-DC, R-DC and F-DC, S-DC and L-DC, S-DC and F-DC, L-DC and F-DC were 0.998, 0.998, 0.998, 0.994, 0.997 and 0.997, respectively (**Supplementary Figure 6**). This analysis showed that the metabolite profiles of the different parts were quite different.

### 3.6 Differential metabolites in R-DC, S-DC, L-DC and F-DC

Out of the 883 metabolites identified, 592 were common across R-Dc, S-Dc, L-Dc, and F-Dc, while R-Dc, S-Dc, L-Dc, and F-Dc each had 16, 0, 5, and 48 unique metabolites, respectively (**Figure 3E**). F-Dc exhibited a higher number of specific metabolites, including 25 flavonoids, 13 phenolic acids, 6 tannins, and others (**Supplementary Table 6**). Particularly noteworthy were Cyanidin-3,5-O-diglucoside (Cyanin), 1-O-Vanilloyl-D-Glucose, 5-Glucosyloxy-2-Hydroxybenzoic acid methyl ester, 2,6-Dimethoxybenzaldehyde, Apigenin-7-O-rutinoside-4’-O-rhamnoside, and several others, which were present in higher concentrations in F-Dc compared to other samples.

The differential metabolites (DAMs) were further filtered using a fold change (FC) threshold of ≥2 or ≤0.5 and a VIP value ≥1. The results of this analysis are depicted in volcano plots (**Supplementary Figure 7**). Between R-Dc and S-Dc, 522 distinct metabolites (260 upregulated) were identified, while between R-Dc and L-Dc, there were 553 distinct metabolites (283 upregulated), and between R-Dc and F-Dc, 592 distinct metabolites (400 upregulated). Between S-Dc and L-Dc, 378 distinct metabolites (232 upregulated) were found. Additionally, between S-Dc and F-Dc, 547 distinct metabolites (394 upregulated) were identified, and between L-Dc and F-Dc, 548 distinct metabolites (378 upregulated) were observed. The classification of metabolites with significant differences in each group highlighted flavonoids, phenolic acids, and lipids as predominant categories.

In comparisons between R-Dc and S-Dc, R-Dc and L-Dc, and R-Dc and F-Dc, there was notable up-regulation of flavonoids and phenolic acids in S-Dc, L-Dc, and F-Dc, with a concurrent down-regulation of lipids. When comparing S-Dc and L-Dc, it was observed that many flavonoids and phenolic acids in L-Dc were up-regulated, while lipids were down-regulated. Similarly, in comparisons between S-Dc and F-Dc, as well as L-Dc and F-Dc, there was significant up-regulation of flavonoids, phenolic acids, and lipid compounds in F-Dc. The analysis of significantly different metabolites between these groups indicated that R-Dc exhibited a higher abundance of lipid differential metabolites, whereas F-Dc showed a greater presence of flavonoid and phenolic acid differential metabolites (**Supplementary Table 7**).

### 3.7 KEGG pathway annotation of the differential metabolites

To investigate the differential metabolites at the gene expression level, we conducted KEGG pathway analysis. The annotation results revealed the involvement of numerous differential metabolites in the biosynthesis of secondary metabolites, phenylpropanoid biosynthesis, and flavonoid biosynthesis. The KEGG pathway enrichment analysis indicated that the primary enrichment of differential metabolites between R-Dc and S-Dc was observed in phenylpropanoid biosynthesis and flavonoid biosynthesis (**Supplementary Figure 8A**). Besides these metabolic pathways, the differential metabolites between R-Dc and L-Dc were also involved in vitamin B6 metabolism, tryptophan metabolism, and plant hormone signal transduction (**Supplementary Figure 8B**). The differential metabolites between R-Dc and F-Dc were primarily associated with biosynthesis of secondary metabolites, flavonoid biosynthesis, and flavone and flavonol biosynthesis (**Supplementary Figure 8C**). The differential metabolites between S-Dc and L-Dc were primarily found in phenylpropanoid biosynthesis, biosynthesis of secondary metabolites, and flavonoid biosynthesis (**Supplementary Figure 8D**). **Supplementary Figure 8E** indicated that the differential metabolites between S-Dc and F-Dc were mainly enriched in phenylpropanoid biosynthesis and galactose metabolism. **Supplementary Figure 8F** shows that the differential metabolites between L-Dc and F-Dc were mainly involved in ABC transporters, flavone and flavonol biosynthesis, flavonoid biosynthesis, and biosynthesis of secondary metabolites. These comparison groups shared several overlapping metabolic pathways, such as the flavonoid biosynthesis pathway, the flavone and flavonol biosynthesis pathway, and the phenylpropanoid biosynthesis pathway. These metabolic pathways are closely related to the investigation of flavonoid’s biological activity.

### 3.8 Key significantly differential metabolites

**Figure 4:**
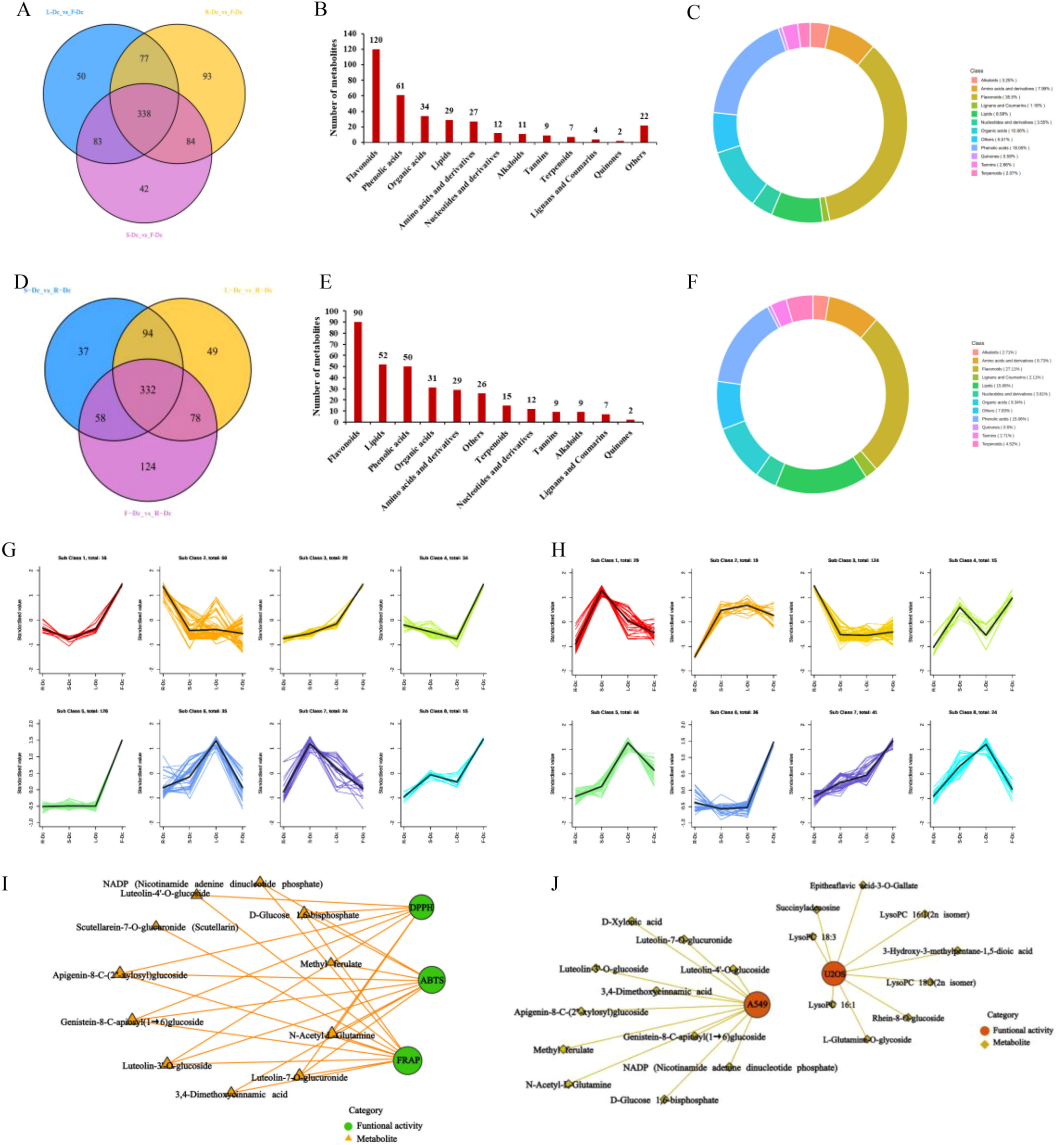
Correlation analysis between biological activity and differential metabolites. **(A)** Venn plot of metabolite differences between R-Dc and F-Dc, S-Dc and F-Dc, L-Dc and F-Dc; **(B)** The proportion of different types of differential metabolites between R-Dc and F-Dc, S-Dc and F-Dc, and L-Dc and F-Dc; **(C)** The number of different types of differential metabolites of R-Dc and F-Dc, S-Dc and F-Dc, L-Dc and F-Dc; **(D)** Venn plot of metabolite differences between S-Dc and R-Dc, L-Dc and R-Dc, F-Dc and R-Dc; **(E)** The proportion of different types of differential metabolites between S-Dc and R-Dc, L-Dc and R-Dc, F-Dc and R-Dc; **(F)** The number of different types of differential metabolites of S-Dc and R-Dc, L-Dc and R-Dc, F-Dc and R-Dc; **(G)** K-means analysis of different metabolites in R-Dc and F-Dc, S-Dc and F-Dc, L-Dc and F-Dc groups; **(H)** K-means analysis of different metabolites in S-Dc and R-Dc, L-Dc and R-Dc, F-Dc and R-Dc groups; **(I)** Correlation analysis between antioxidant activity and differential metabolites; **(J)** Correlation analysis between antitumor activity and differential metabolites.

F-Dc was chosen as the core group, and the differential metabolites between different tissues were screened for analysis using a fold change (FC) ≥2 or (FC) ≤0.5 and a VIP value ≥1. Venn diagrams were constructed to compare the differential metabolites between R-Dc and F-Dc, S-Dc and F-Dc, and L-Dc and F-Dc. A total of 338 differential metabolites overlapped between R-Dc and F-Dc, S-Dc and F-Dc, and L-Dc and F-Dc (**Figure 4A**). The 338 overlapping differential metabolites are considered key metabolites influencing the production of various biological activities between F-Dc and other tissues (R-Dc, S-Dc, L-Dc). They are categorized into 12 different classes, which include 120 flavonoids, 61 phenolic acids, 34 organic acids, 29 lipids, 27 amino acids and their derivatives, 12 nucleotides and their derivatives, 11 alkaloids, 9 tannins, 7 terpenoids, 4 lignans and coumarins, 2 quinones, and 10 other classes (**Figure 4B**). Flavonoids accounted for 35.5% of the key differential metabolites, while phenolic acids accounted for 18.05% (**Figure 4C**).

We selected R-Dc as the core group and constructed Venn diagrams for differential metabolites between S-Dc and R-Dc, L-Dc and R-Dc, and F-Dc and R-Dc. We found a total of 332 overlapping differential metabolites among the three groups (**Figure 4D**). These metabolites are considered to be key factors influencing the different biological activities between R-Dc and other tissues (S-Dc, L-Dc, F-Dc). It is divided into 12 different categories, including 90 flavonoids, 52 lipids, 50 phenolic acids, 31 organic acids, 29 amino acids and derivatives, 15 terpenes, 12 nucleotides and derivatives, 9 tannins, 9 alkaloids, 7 lignans and coumarins, 2 quinones, and 26 other categories (**Figure 4E**). Among them, flavonoids, lipids, and phenolic acids account for 27.11%, 15.66%, and 15.06% of the key differential metabolites, respectively (**Figure 4F**).

In order to clarify the relative content variation trends of key differential metabolites in different tissues, the relative content of 338 overlapping differential metabolites found in the F-Dc core group and 332 overlapping differential metabolites found in the R-Dc core group were standardized using Z-score. Subsequently, a K-means analysis (**Figures 4G, H**) was performed.

### 3.9 Correlation analysis of key significantly different metabolites with antioxidant and antitumor activities

The K-means analysis of differential metabolites in the F-Dc core group revealed that the relative content of 16 differential metabolites in the first subclass correlated with the antioxidant activity trend in various carnation parts. A correlation analysis was conducted between three antioxidant methods and 16 differentially expressed metabolites, revealing a complex relationship (**Figure 4I**) between 11 metabolites and antioxidant activity. Two phenolic acids and six flavonoid compounds, as well as secondary metabolites such as methyl ferulate, luteolin -3’ -*O*-glucoside, luteolin -4’-*O*-glucoside, genistein 8-C-apiosyl (1→6) glucoside, and apigenin 8-C- (2”-xylosyl) glucoside, were found in high concentrations, suggesting their potential as antioxidants.

The relative abundance of 16 differential metabolites in the first subclass of K-means analysis, with F-Dc as the core group, was found to be consistent with the inhibitory effect of different parts of carnation on A549 cells. A correlation analysis was performed between these two factors, and the results show that 11 metabolites are correlated with anti-tumor activity (**Figure 4J**).

In the K-means analysis of differential metabolites with R-Dc as the core group, the third subclass consists of 124 differential metabolites. The relative abundance of these metabolites in different tissues is consistent with the inhibition trend of different tissues on U2OS cells. Fifty differential metabolites with high abundance in R-Dc were selected for correlation analysis. The results indicate that 9 differential metabolites may affect the inhibition of U2OS cells (**Figure 4J**). Secondary metabolites form the basis of functional activities in plant medicine. Among the secondary metabolites with high abundance and anti-tumor activity are rhein-8-*O*-glucoside and epitheaflavic acid-3-*O*-gallate.

### 3.10 Separation and purification of compounds from carnation

**Figure 5:**
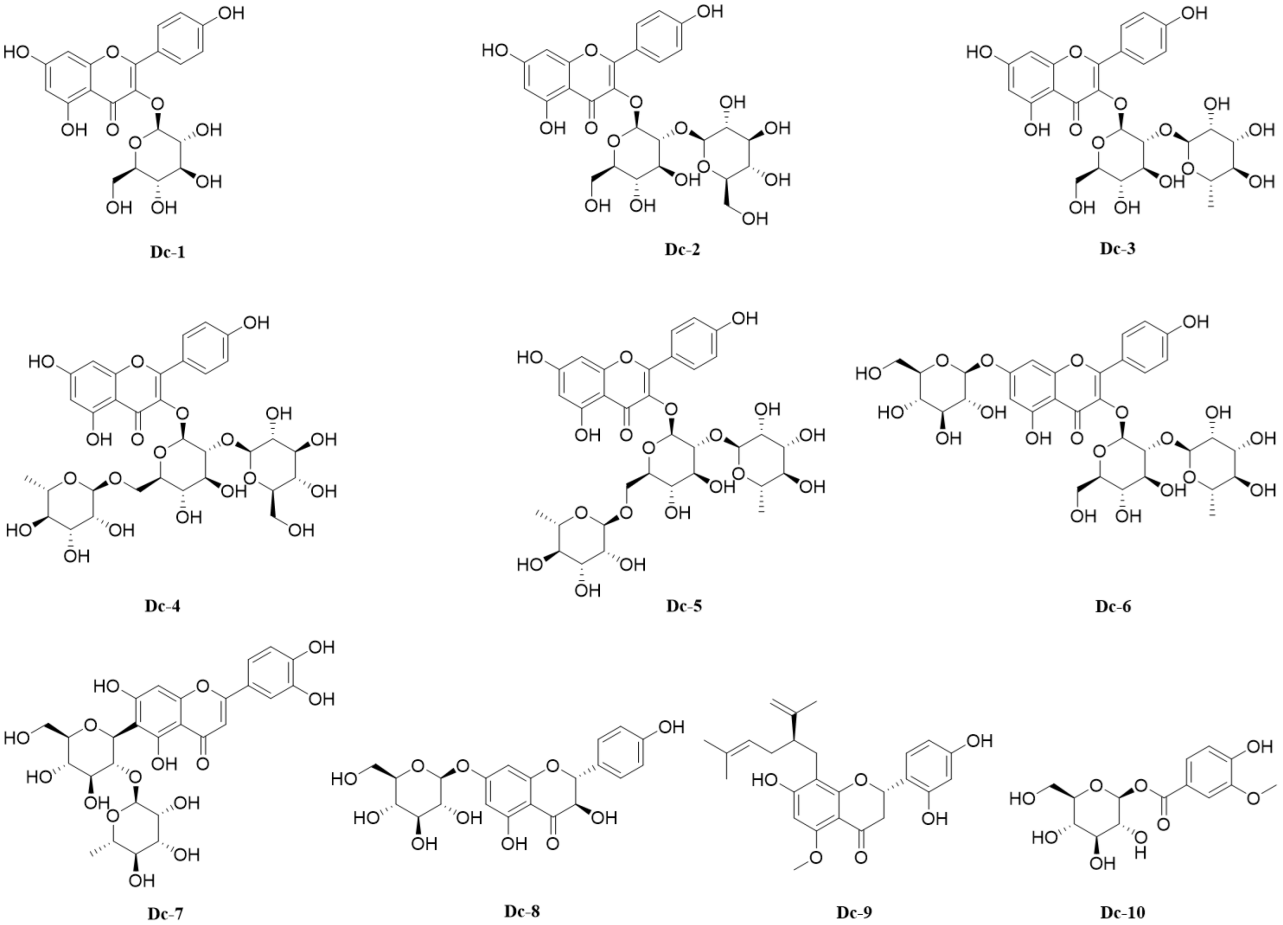
10 compounds isolated from the carnation flowers.

A variety of spectral techniques were used to analyze the compounds isolated and purified from the carnation, a comparison of the literature data identified 10 compounds (**Supplementary File 1**), including 9 flavonoids and 1 phenolic glycoside. Compounds 1–6 are flavonol glycosides, compound 7 is flavone glycosides, compound 8 is dihydroflavonol glycosides, compound 9 is flavanones. The 10 compounds were: Astragalin (Dc-1), Kaempferol 3-*O*-sophoroside (Dc-2), Kaempferol 3-neohesperidoside (Dc-3), Kaempferol 3-*O*-(2’’-glucosyl) rutinoside (Dc-4), Clitorin (Dc-5), Kaempferol 3-neohesperidoside 7-glucoside (Dc-6), Isoorientin 2’’-*O*-rhamnoside (Dc-7), Sinensin (Dc-8), Kurarinone (Dc-9), 1-*O*-Vanilloylglucose (Dc-10). The chemical structure of compounds 1–10 is shown in **Figure 5**.

### 3.11 Screening results of antioxidant activity of compounds

**Figure 6:**
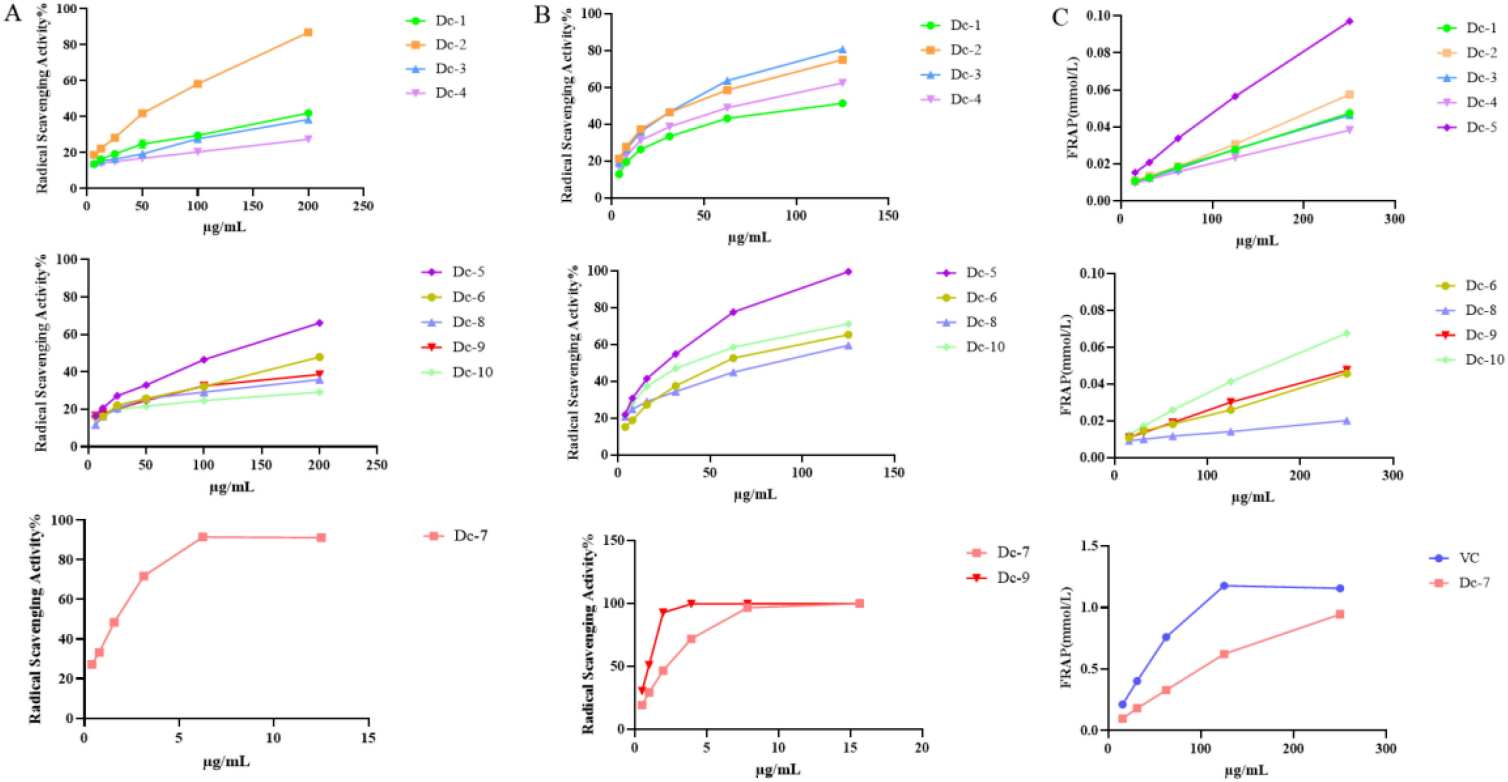
Antioxidant capacity of the Dc-1~ Dc-10 compound. **(A)** DPPH free radical scavenging rate; **(B)** ABTS free radical scavenging rate; **(C)** Ferric ion reducing antioxidant power (FRAP).

**Figure 6A** illustrates that Dc-7 exhibits the highest capacity for scavenging DPPH free radicals, followed by Dc-2 and Dc-5, whereas the remaining seven monomers demonstrate poor scavenging abilities. Within the experimental concentration range, the scavenging effect of the ten compounds on DPPH free radicals increased as the sample concentration increased.

**Figure 6B** demonstrates that all ten compounds exhibit the ability to scavenge ABTS free radicals. Dc-7 and Dc-9 exhibited the highest efficiency in scavenging ABTS free radicals when the mass concentration exceeded 5 μg/mL. Additionally, the remaining eight compounds demonstrated ABTS free radical scavenging ability, which further improved with increasing mass concentration.

**Supplementary Table 8** reveals that, apart from Dc-7, the EC_50_ values of the remaining nine compounds for ABTS were lower than those for DPPH, suggesting superior scavenging effects of all nine compounds on ABTS free radicals compared to DPPH free radicals. Among the ten samples, only Dc-2, Dc-5, and Dc-7 exhibited scavenging effects on both DPPH and ABTS free radicals within the experimental concentration range, while the other seven samples demonstrated scavenging effects solely on ABTS free radicals. Dc-9 exhibited an ABTS EC_50_ value of 0.824 ± 0.005 µg/mL, indicating the most efficient scavenging effect on the free radicals. The mean EC_50_ value was 0.549 ± 0.016 µg/mL, which closely approximated the positive control (VC) value.

**Figure 6C** presents the total antioxidant capacity of Dc-1 to Dc-10 at various concentrations. These results demonstrate that Dc-7 exhibits the strongest ferric ion reducing antioxidant power at the same concentration, reaching 81.77% of that of the positive control (VC) at a concentration of 250 μg/mL. Among the remaining nine compounds, the Dc-5 compound exhibited the strongest ferric ion reducing antioxidant power, followed by the Dc-10 compound. The reduction capacity of Dc-1~Dc-10 increases with the rise in mass concentration within the concentration range of 15.625 ~ 250 μg/mL.

At the experimental concentration, Dc-2, Dc-5, and Dc-7 demonstrated scavenging effects on both DPPH and ABTS free radicals, with the scavenging effect being concentration-dependent. Dc-7 exhibits the lowest EC_50_ value for both DPPH and ABTS, indicating its strong ability to scavenge free radicals. Additionally, it demonstrates a high capacity to reduce iron ions, making it the most effective antioxidant. Dc-1, Dc-3, Dc-4, Dc-6, Dc-8, Dc-9, and Dc-10 exhibit a significant scavenging effect on ABTS free radicals, but not on DPPH. They also display a relatively low reducing capacity for iron ions. This may be attributed to the variation in antioxidant mechanisms of different substances across different antioxidant assessment systems (Gulcin, 2020).

### 3.12 Molecular docking result

**Figure 7:**
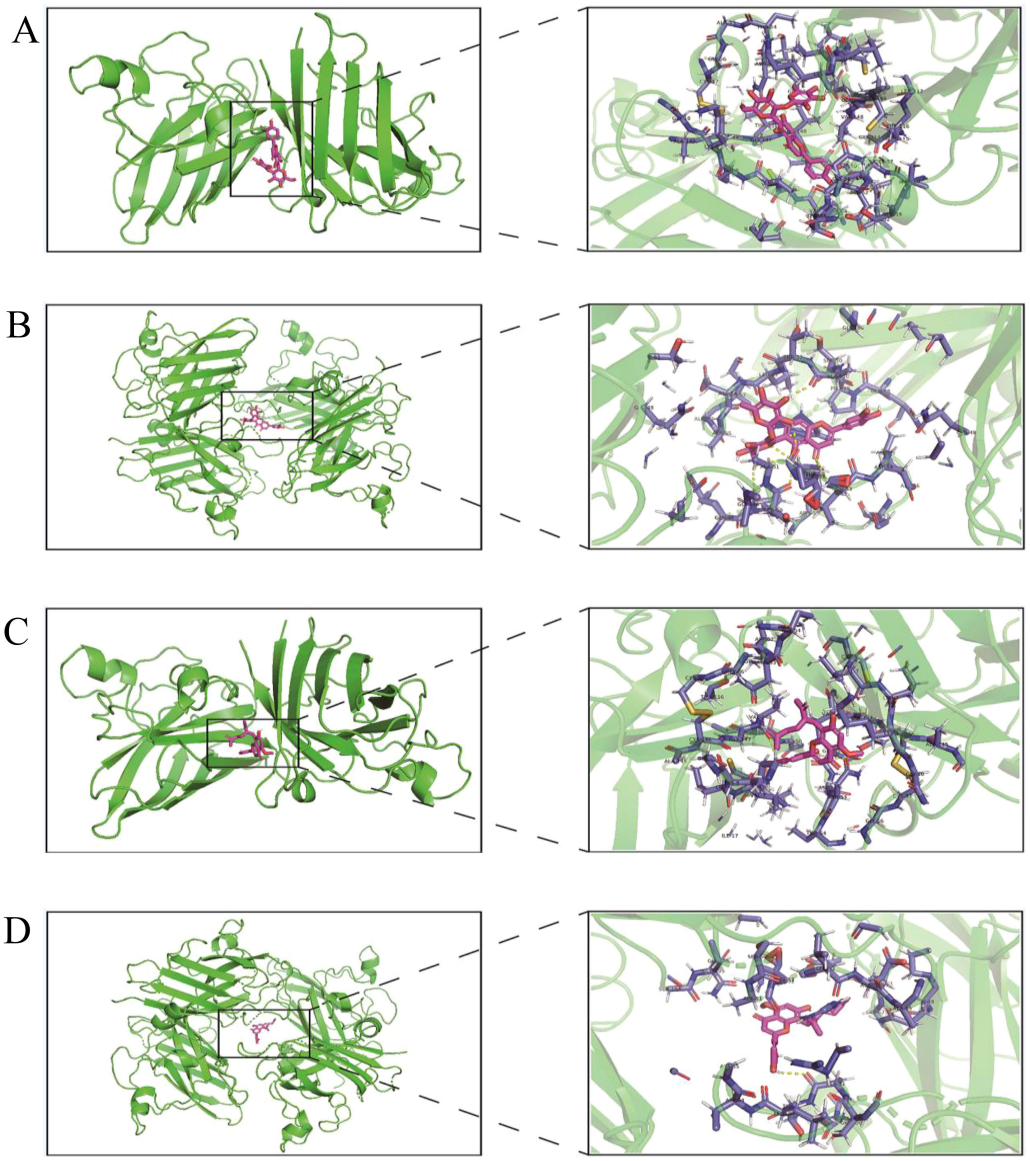
Virtual docking of Isoorientin 2”-*O*-rhamnoside and Kurarinone to oxidation related genes. **(A)**Virtual docking between SOD1 and Isoorientin 2”-*O*-rhamnoside; **(B)** Virtual docking of SOD3 with Isoorientin 2”-*O*-rhamnoside has been described; **(C)** Virtual interconnector between SOD1 and Kurarinone; **(D)** Virtual interconnector between SOD3 and Kurarinone.

The protein structures of 431 oxidation-related genes and 415 oxidation-associated genes were identified. Based on the experimental findings, we found that two monomers, Isoorientin 2”-*O*-rhamnoside and Kurarinone, with strong antioxidant capacity, were associated with the oxidation-related gene set and divided into two docking cohorts. The binding strength between the drug and protein structure was quantified using the docking binding energy. Lower binding energy facilitated easier bonding between the two. The results were visualized using the open-source version of Pymol software. The docking results revealed that the interaction between Isoorientin 2”-*O*-rhamnoside and SOD1 and SOD3, members of the SOD family of antioxidant enzymes, was highly favorable. The docking energies for SOD1 and SOD3 were determined to be -13.20 kcal/mol and -10.43 kcal/mol, respectively (**Figures 7A, B**). Additionally, the docking energies of Kurarinone with SOD1 and SOD3 aligned with our expectations, measuring -10.15 kcal/mol and -7.78 kcal/mol, respectively (**Figures 7C, D**).

## 4 Discussion

The experimental findings reveal that the levels of polyphenols and flavonoids in different parts of Dianthus caryophyllus correlate with their antioxidant capacity and their ability to inhibit A549 cells. These secondary metabolites exhibit potent biological activities. For instance, luteolin, a flavonoid compound, has shown significant tumor-inhibitory effects on non-small-cell lung cancer (Jiang et al., 2018). Genistein, an isoflavone compound, demonstrates preventive properties against prostate cancer, breast cancer, and other malignancies (Ko, 2014). Methyl ferulate, a phenolic acid compound, exhibits notable antioxidant, antibacterial, and anti-inflammatory activities (Kikuzaki et al., 2002; Li et al., 2021; Phuong et al., 2014). The presence of abundant flavonoid and phenolic acid compounds in Dianthus caryophyllus flowers suggests that the flowers may possess significant biological activity.

To identify metabolites in different tissue parts of Dianthus caryophyllus and elucidate the material basis for varying antioxidant activity among its organs, we conducted comprehensive targeted metabolomics analysis using UPLC-MS/MS on the roots, stems, leaves, and flowers of carnations. A total of 883 metabolites were identified, predominantly comprising flavonoids, phenolic acids, and lipids. Principal Component Analysis (PCA), Hierarchical Cluster Analysis (HCA), and Orthogonal Partial Least Squares Discriminant Analysis (OPLS-DA) highlighted significant differences in metabolite composition across these tissue parts, suggesting these variations could underlie distinct functional activities in specific locations. Furthermore, KEGG pathway analysis revealed that these metabolites are primarily involved in biosynthetic pathways of flavonoids, flavones, flavonols, and phenylpropanoids. These pathways directly relate to the exploration of biological activities exhibited by the principal flavonoids present in carnation.

Correlation analysis between selected metabolites and the antioxidant activities of different parts of carnation revealed a potential association between the higher antioxidant capacity and anti-tumor activity of carnation flowers, attributed to the abundant presence of the active compound methyl ferulate (Kikuzaki et al., 2002). Flavonoids are predominantly found as glycosides, such as luteolin-3’-O-glucoside, genistein 8-C-apiosyl (1C-a glucoside, apigenin 8-C-(2”-xylosyl) glucoside, among others. R-Dc exhibits stronger inhibitory effects on U2OS osteosarcoma cells, possibly due to its elevated content of anthraquinone compounds like rhein-8-O-glucoside. Anthraquinones have been historically utilized in treating various conditions, including tumors, viral diseases, and inflammation (Cheng et al., 2021).

Carnation is rich in flavonoids, flavonoids are an important natural organic compound with a high degree of chemical reactivity, which can scavenge free radicals from organisms and exert antioxidant effects (Wang et al., 2022). Kaempferol and quercetin are recognized as prominent flavonoid antioxidants (Xu et al., 2019; Kluska et al., 2022). Additionally, Farrerol and flavonol glycosides are frequently employed as antioxidants (Caddeo et al., 2019). The extraction, separation, and structural identification of the chemical constituents of carnation flowers led to the identification of 10 compounds, 9 of which were flavonoids. To compare the variations in antioxidant activities among the different flavonoids, the antioxidant activities of these compounds were evaluated.

The structure-activity relationship of flavonoids in relation to oxidation was investigated based on experimental findings. All nine flavonoids contained phenolic hydroxyl groups, each with varying numbers and locations. The experimental findings indicated that the antioxidant activity of the flavonoids was associated with the number of phenol hydroxyl groups in the structure. Among the flavonoids tested, Isoorientin 2”-*O*-rhamnoside, which contained 4 phenolic hydroxyl groups, demonstrated the most prominent antioxidant effect. This could be attributed to the ability of the hydroxyl group in the phenol to react with oxygen’s free radicals, resulting in the formation of relatively stable semiquinone radicals. These radicals then interrupt the free-radical chain reaction, leading to the antioxidant activity exhibited (Ravishankar et al., 2013). Additionally, the experiment revealed that both Isoorientin 2”-*O*-rhamnoside and Kurarinone possessed two phenolic hydroxyl groups on the B benzene ring. This observation suggests that the hydroxyl group on the B benzene ring in the flavonoid structure exhibits a higher activity compared to that of the A ring, which corroborates existing literature (Foti and Ruberto, 2001).

The primary role of SOD is to catalyze the disproportionation of superoxide anion radicals into hydrogen peroxide and oxygen molecules. The production of superoxide anion radicals is a normal metabolic process in the body. However, the accumulation of free radicals can lead to peroxidation of cell membrane lipids, causing membrane fission, cell damage, and potential fatality (Bastin et al., 2023). SODs play a pivotal role as highly effective scavengers of free radicals in the body, thereby sustaining the metabolic balance of the organism. The imbalance between oxidation and antioxidants can be rectified by utilizing natural antioxidants derived from plants. Molecular docking was employed to confirm the presence of two compounds exhibiting strong antioxidant activity. The study revealed that both Isoorientin 2”-*O*-rhamnoside and Kurarinone displayed optimal binding affinity to SOD1 and SOD3 proteins.

## 5 Conclusion

In summary, this study conducted a detailed study on the chemical composition of carnation roots, stems, leaves, and flowers, highlighting their role as the basis for antioxidant and anti-tumor activities. Our findings showed that methanol extracts from carnation flowers and roots contained potent antioxidants such as methyl ferulate and luteolin-4’-O-glucoside. Extracts R-Dc and F-Dc from carnation significantly induced apoptosis in tumor cells. Additionally, anthraquinone compounds like rhein-8-O-glucoside in carnation roots may enhance their antioxidant properties. We identified and isolated 10 different compounds from carnation, including iso-orientin 2’-O-rhamnoside and Kurarinone, which exhibited significant antioxidant activity. These compounds were validated for their binding affinity with SOD1 and SOD3 through antioxidant screening and molecular docking. This study provides a theoretical basis for the comprehensive utilization and development of carnation components.

## Data Availability

The original contributions presented in the study are included in the article/**Supplementary Material**. Further inquiries can be directed to the corresponding authors.
