# Supplementary material for: Study on chemical constituents and antioxidant activities of Dianthus caryophyllus L
Source: Front Plant Sci. 2024 Aug 22;15:1438967. doi: 10.3389/fpls.2024.1438967 (PMC11374617; doi:10.3389/fpls.2024.1438967)
Supplement: Supplementary file 1 [file DataSheet1.docx]

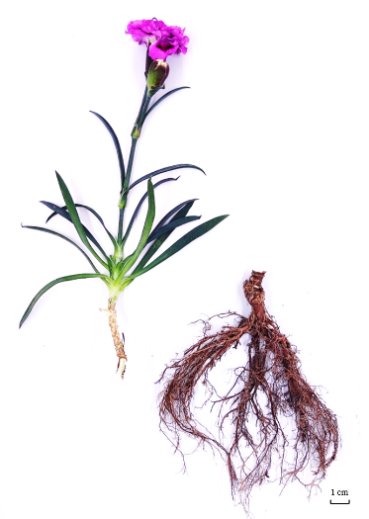


**Supplementary Fig. 1.** The roots, stems, leaves, and flowers of *Dianthus caryophyllus* L.


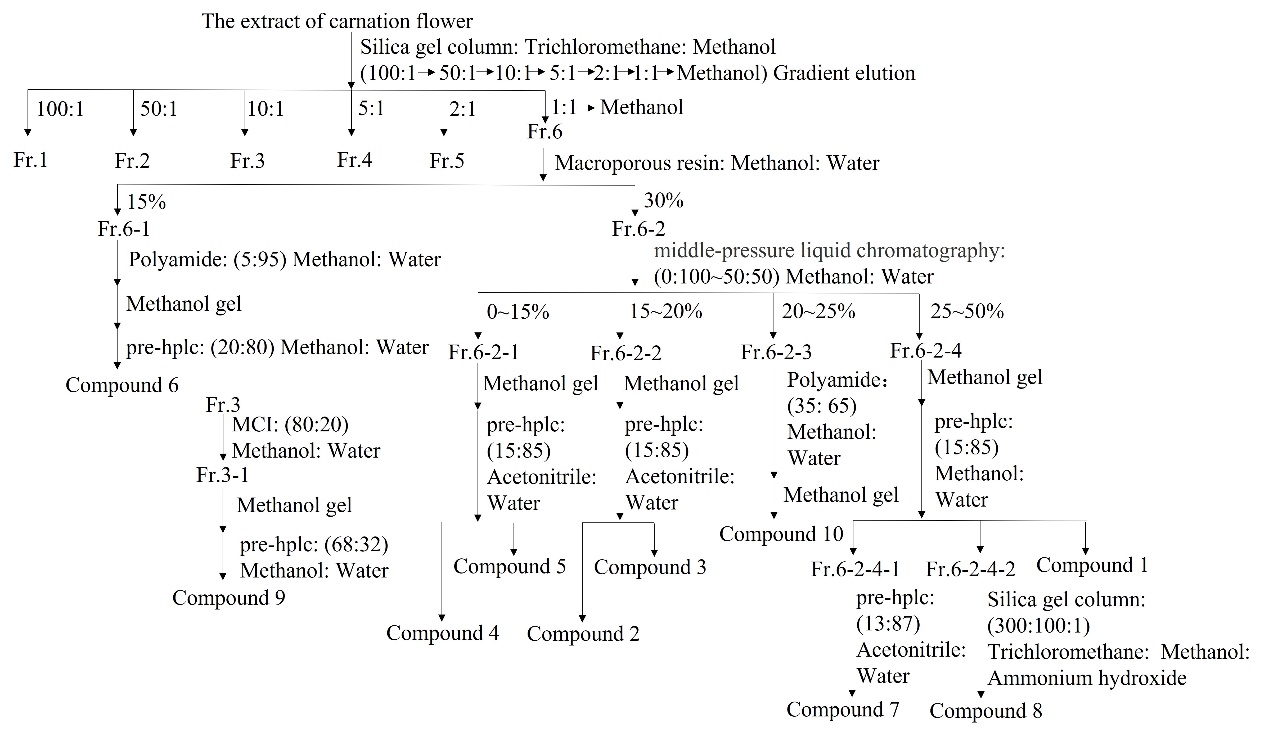


**Supplementary Fig. 2.** Schematic diagram of separation process of extracts of Carnation flower.


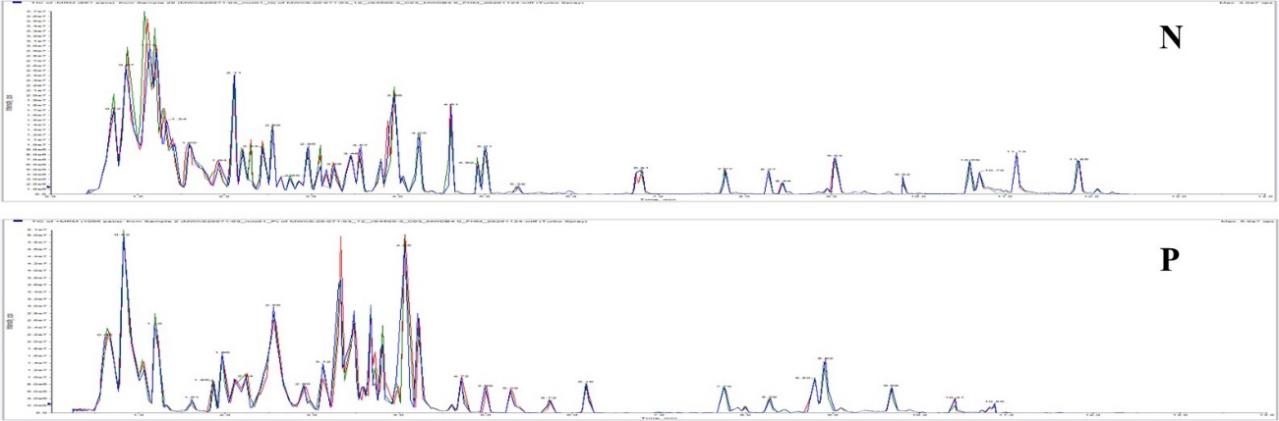


**Supplementary Fig.3.** Total ion chromatogram of Carnation mixed quality spectrum analysis.

Note: N represents negative ion mode, P represents positive ion mode.


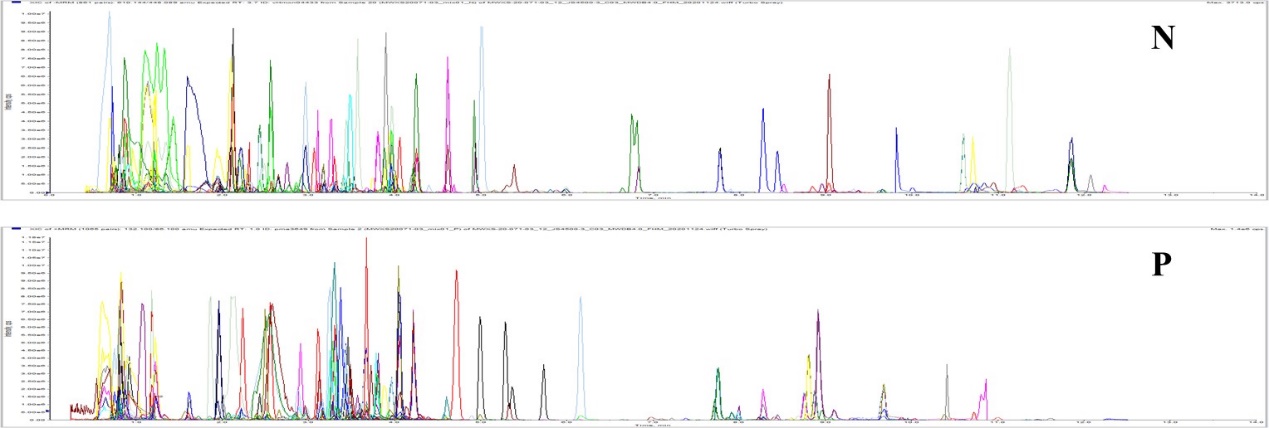


**Supplementary Fig.4.** Multimodal map of MRM metabolites of Carnation.

Note: N represents negative ion mode, P represents positive ion mode.


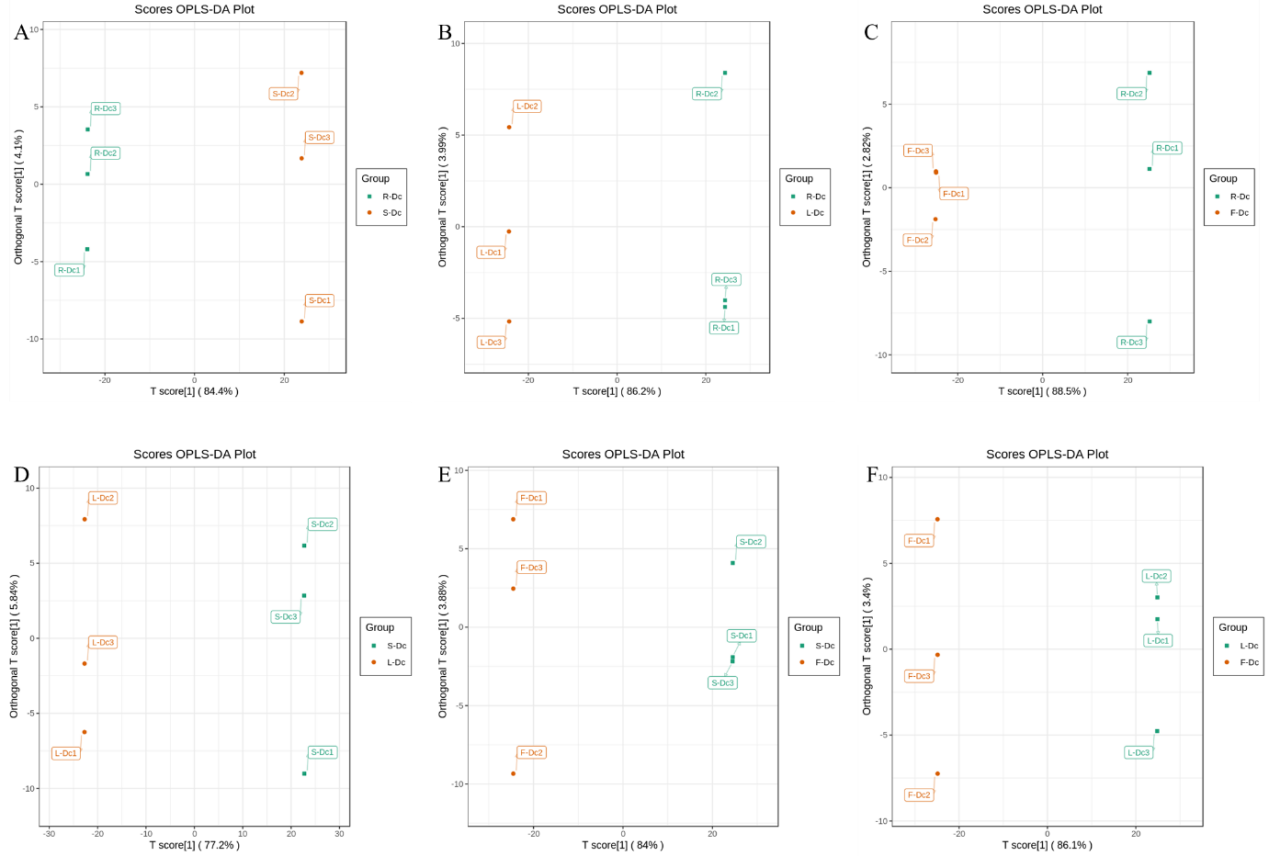


**Supplementary Fig.5.** The score plots of OPLS-DA pairwise comparisons of metabolites between: (A) R-DC and S-DC; (B) R-DC and L-DC; (C) R-DC and F-DC; (D) S-DC and L-DC; (E) S-DC and F-DC; and (F) L-DC and F-DC.

**
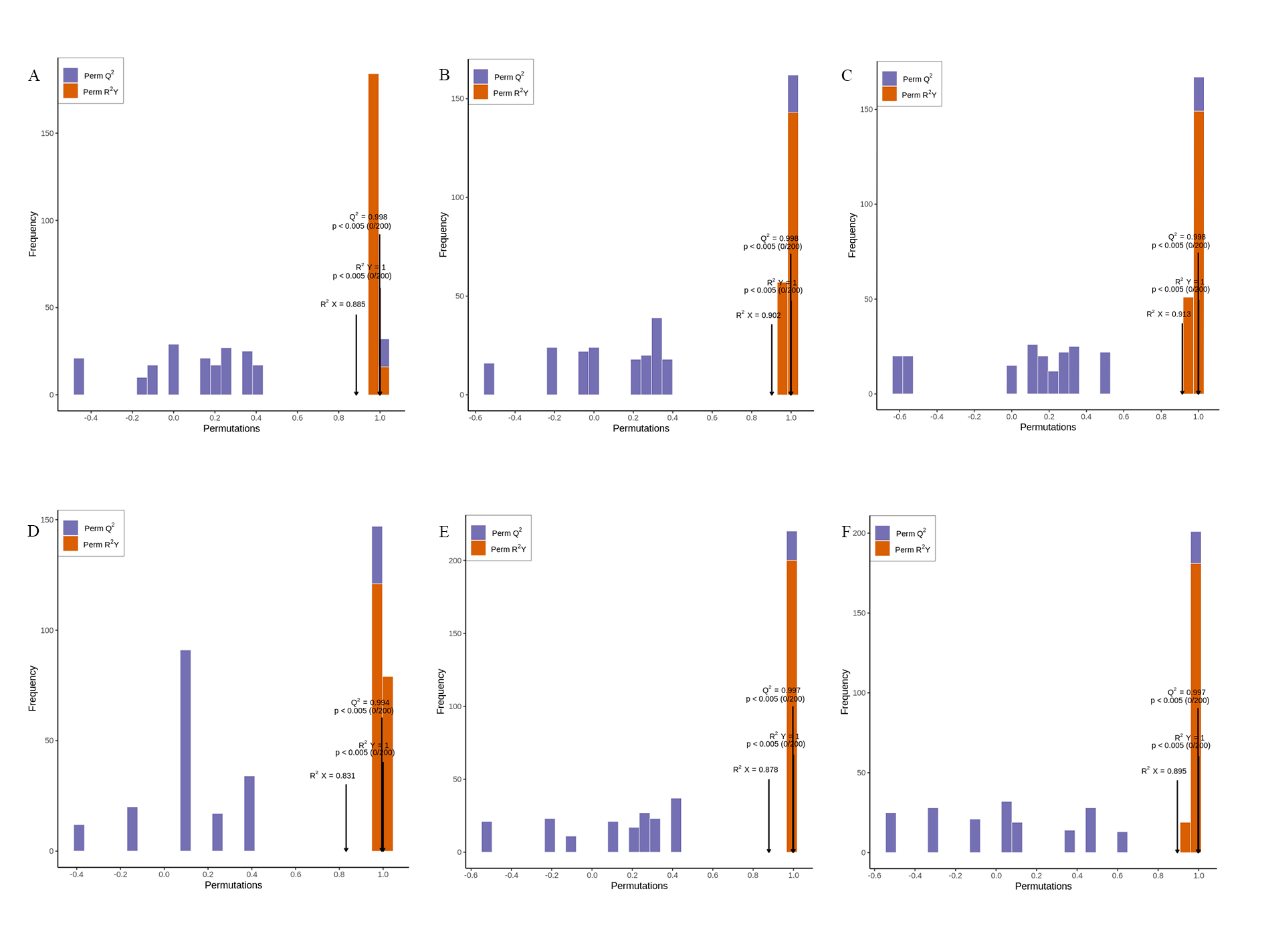
** **Supplementary Fig. 6.** OPLS-DA verification diagram of the pairwise comparison of metabolites between: (A) R-DC and S-DC; (B) R-DC and L-DC; (C) R-DC and F-DC; (D) S-DC and L-DC; (E) S-DC and F-DC; (F) L-DC and F-DC.


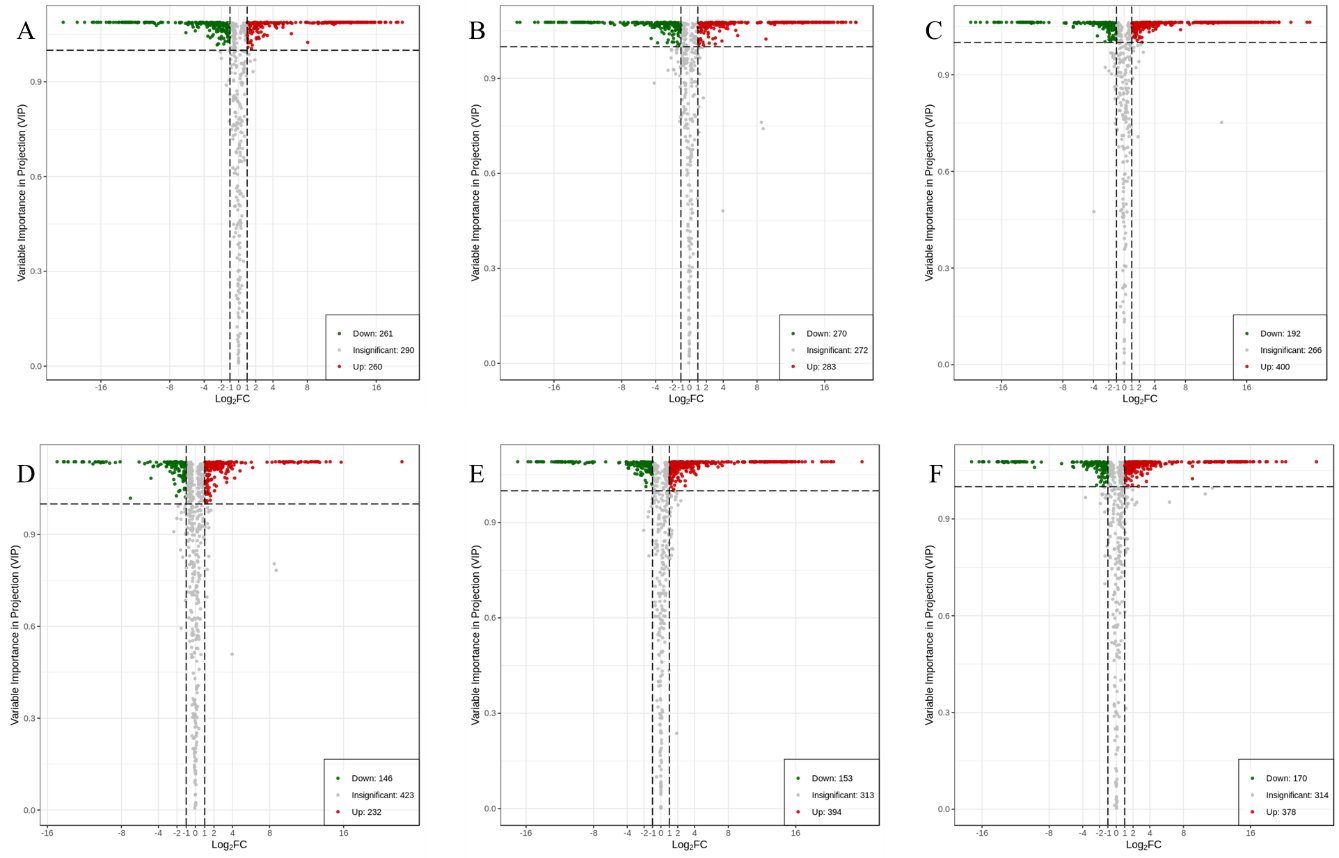


**Supplementary Fig. 7.** Volcano plots of differential metabolites in the pairwise comparison between: (A) R-DC and S-DC; (B) R-DC and L-DC; (C) R-DC and F-DC; (D) S-DC and L-DC; (E) S-DC and F-DC; (F) L-DC and F-DC.

Note: Each point in the volcano map represents a metabolite, the abscissa represents the logarithmic value of the difference of relative content of a certain metabolite in the two samples, the ordinate represents the VIP value. The greater the absolute value of the abscissa, the greater the multiple difference in the expression level between the two samples; the greater the ordinate value, the more significant the differential expression, and the more reliable the differentially expressed metabolites screened. In the figure, the green dots represent down-regulated differentially metabolites, the red dots represent up-regulated differentially metabolites, and gray represents detected but not significantly different metabolites.


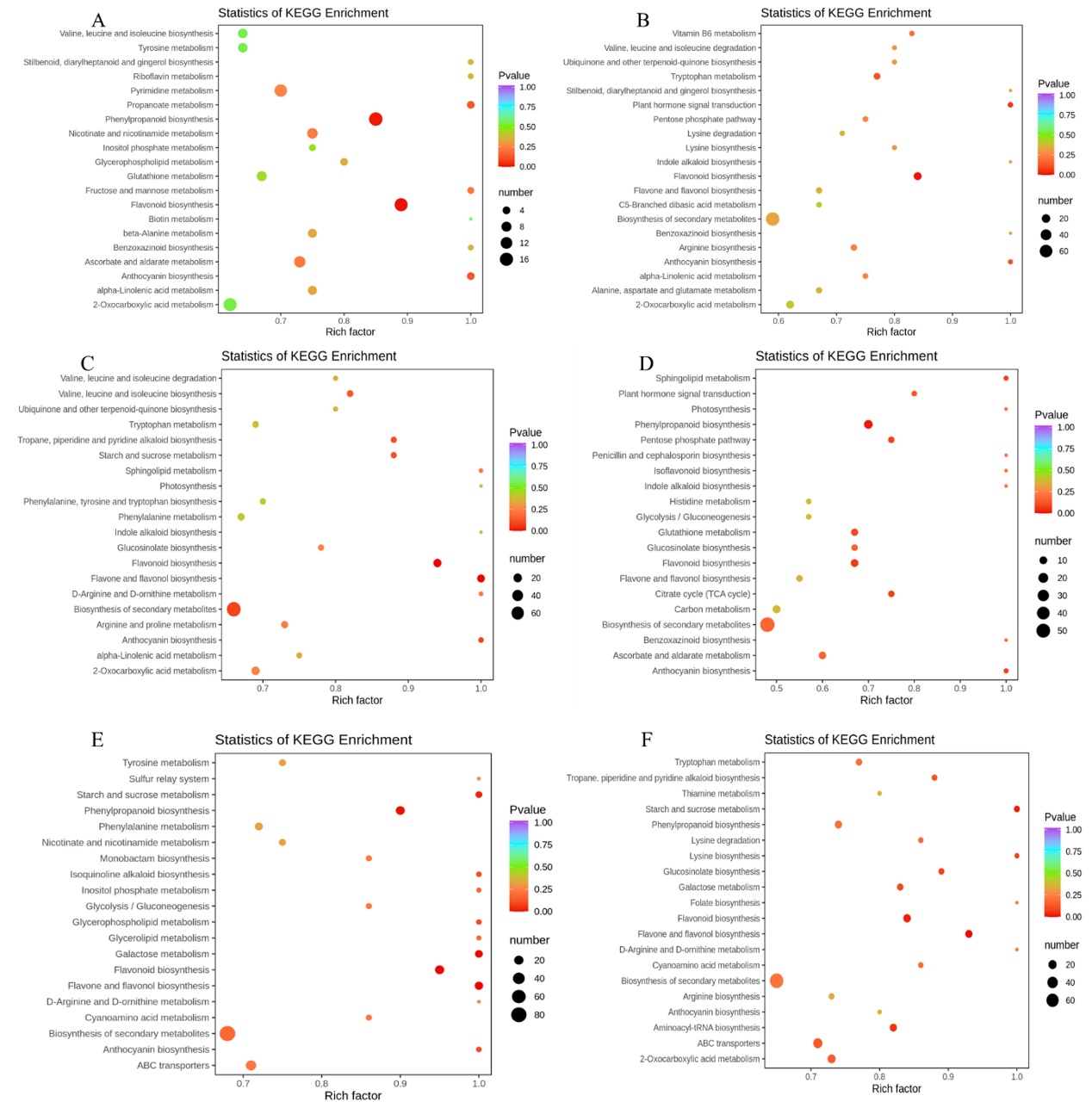
 **Supplementary Fig. 8.** KEGG annotations and enrichment results of the differentially expressed metabolites in the pairwise comparison between: (A) R-Dc and S-Dc; (B) R-Dc and L-Dc; (C) R-Dc and F-Dc; (D) S-Dc and L-Dc; (E) S-Dc and F-Dc; (F) L-Dc and F-Dc.
