# Supplementary material for: Study on chemical constituents and antioxidant activities of Dianthus caryophyllus L
Source: Front Plant Sci. 2024 Aug 22;15:1438967. doi: 10.3389/fpls.2024.1438967 (PMC11374617; doi:10.3389/fpls.2024.1438967)
Supplement: Supplementary file 2 [file DataSheet2.docx]

**Supplementary file: Compounds spectral analysis.**

Compound 1 was identified as Astragalin based on ^1^H-NMR, ^13^C-NMR data and references( Okuyama et al., 1978). Astragalin (**1**): yellow amorphous powder, ESI *m*/*z* 471 [M + Na]^+^, C_21_H_20_O_11_. ^1^H-NMR (500 MHz, CD_3_OD) *δ*: 8.05 (2H, d, *J* = 8.9 Hz, H-2', 6'), 6.88 (2H, d, *J* = 8.9 Hz, H-3', 5'), 6.39 (1H, d, *J* = 2.1 Hz, H-8), 6.19 (1H, d, *J* = 2.1 Hz, H-6), 5.24 (1H, d, *J* = 7.5 Hz, H-1''), 3.68 (1H, dd, *J* = 11.9, 2.4 Hz, H-6''a), 3.52 (1H, dd, *J* = 11.9, 5.5 Hz, H-6''b), 3.19 (1H, m, H-5'') (Fig. 1). ^13^C-NMR (125 MHz, DMSO-*d*_6_) *δ*: 156.3 (C-2, 9), 133.2 (C-3), 177.5 (C-4), 161.2 (C-5), 98.7 (C-6), 164.1 (C-7), 93.7 (C-8), 104.0 (C-10), 120.9 (C-1'), 130.9 (C-2', 6'), 115.1 (C-3', 5'), 159.9 (C-4'), 100.8 (C-1''), 74.2 (C-2''), 77.5 (C-3''), 69.9 (C-4''), 76.4 (C-5''), 60.8 (C-6'') (Fig. 2).

Compound 2 was identified as Kaempferol 3-*O*-sophoroside based on ^1^H-NMR, ^13^C-NMR data and references( Wolfram et al., 2010). Kaempferol 3-*O*-sophoroside (**2**): yellow amorphous powder, ESI *m*/*z* 633 [M + Na]^+^, C_27_H_30_O_16_. ^1^H-NMR (500 MHz, DMSO-*d*_6_) *δ*: 12.64 (1H, s, 5-OH), 8.03 (2H, d, *J* = 8.9 Hz, H-2', 6'), 6.90 (2H, d, *J* = 8.9 Hz, H-3', 5'), 6.41 (1H, d, *J* = 2.0 Hz, H-8), 6.17 (1H, d, *J* = 2.0 Hz, H-6), 5.68 (1H, d, *J* = 7.2 Hz, H-1''), 4.60 (1H, d, *J* = 7.8 Hz, H-1''') (Fig. 3). ^13^C-NMR (125 MHz, DMSO-*d*_6_) *δ*: 158.8 (C-2, 9), 134.9 (C-3), 179.6 (C-4), 163.2 (C-5), 100.0 (C-6), 166.3 (C-7), 94.8 (C-8), 105.7 (C-10), 122.8 (C-1'), 132.3 (C-2', 6'), 116.2 (C-3', 5'), 161.4 (C-4'), 101.0 (C-1''), 82.5 (C-2''), 78.3, 78.2 (C-3'', 3'''), 71.3(C-4''), 77.9(C-5'', 5'''), 62.6(C-6''), 104.7 (C-1'''), 75.5 (C-2'''), 71.1 (C-4'''), 62.4 (C-6''') (Fig. 4).

Compound 3 was identified as Kaempferol 3-neohesperidoside based on ^1^H-NMR, ^13^C-NMR data and references( Nørbaek & Kondo, 1999). Kaempferol 3-neohesperidoside (**3**): yellow amorphous powder, ESI-MS *m/z* 617 [M + Na]^+^, C_27_H_30_O_15_. ^1^H-NMR (500 MHz, DMSO-*d*_6_) *δ*: 8.02 (2H, d, *J* = 8.9 Hz, H-2', 6'), 6.87 (2H, d, *J* = 8.9 Hz, H-3', 5'), 6.39 (1H, d, *J* = 2.1 Hz, H-8), 6.16 (1H, d, *J* = 2.1 Hz, H-6), 5.65 (1H, d, *J* = 7.4 Hz, H-1''), 5.22 (1H, d, *J* = 1.6 Hz, H-1''') (Fig. 5). ^13^C-NMR (125 MHz, DMSO-*d*_6_) *δ*: 159.9 (C-2), 132.7 (C-3), 177.3 (C-4), 161.2 (C-5), 98.3 (C-6), 163.6 (C-7), 93.8 (C-8), 156.4 (C-9), 103.7 (C-10), 120.9 (C-1'), 130.8 (C-2', 6'), 155.9 (C-4'), 115.1 (C-3', 5'), 99.0 (C-1''), 77.6 (C-2''), 77.5 (C-3''), 70.2 (C-4''), 77.3 (C-5''), 60.8 (C-6''), 100.6 (C-1'''), 70.6 (C-2'''), 70.2 (C-3'''), 71.9 (C-4'''), 68.3 (C-5'''), 17.6 (C-6''') (Fig. 6).

Compound 4 was identified as Kaempferol 3-*O*-(2''-glucosyl)rutinoside based on ^1^H-NMR, ^13^C-NMR data and references(Budzianowski, 1990). Kaempferol 3-*O*-(2''-glucosyl) rutinoside (**4**): yellow amorphous powder, ESI-MS *m/z* 779 [M + Na]^+^, C_33_H_40_O_20_. ^1^H-NMR (500 MHz, DMSO-*d*_6_) *δ*: 7.97 (2H, d, *J* = 8.9 Hz, H-2', 6'), 6.88 (2H, d, *J* = 8.9 Hz, H-3', 5'), 6.38 (1H, d, *J* = 2.1 Hz, H-8), 6.17 (1H, d, *J* = 2.1 Hz, H-6), 5.52 (1H, d, *J* = 6.9 Hz, H-1''), 4.57 (1H, d, *J* = 7.8 Hz, H-1'''), 4.35 (1H, d, *J* = 1.2 Hz, H-1''''), 0.92 (3H, d, *J* = 6.1 Hz, H-CH_3_) (Fig. 7). ^13^C-NMR (125 MHz, DMSO-*d*_6_) *δ*: 156.4 (C-2), 132.8 (C-3), 177.4 (C-4), 161.2 (C-5), 98.2 (C-6), 164.1 (C-7), 93.7 (C-8), 156.3 (C-9), 103.9 (C-10), 120.9 (C-1'), 131.0 (C-2', 6'), 160.0 (C-4'), 115.2 (C-3', 5'), 98.2 (C-1''), 82.3 (C-2''), 76.6 (C-3''), 70.2 (C-4''), 69.7 (C-5''), 66.0 (C-6''), 103.9 (C-1'''), 74.3 (C-2'''), 76.4 (C-3'''), 70.6 (C-4'''), 77.1 (C-5'''), 60.8 (C-6'''), 100.4 (C-1''''), 70.3 (C-2''''), 69.5 (C-3''''), 71.8 (C-4''''), 68.1 (C-5''''), 17.6 (C-6'''') (Fig. 8).

Compound 5 was identified as Clitorin based on ^1^H-NMR, ^13^C-NMR data and references(Kazuma, Noda, & Suzuki, 2003). Clitorin (**5**): yellow amorphous powder, ESI-MS *m/z* [M + Na]^+^, C_30_H_40_O_19_. ^1^H-NMR (500 MHz, CD_3_OD) *δ*: 8.02 (2H, d, *J* = 8.9 Hz, H-2', 6'), 6.90 (2H, d, *J* = 8.9 Hz, H-3', 5'), 6.39 (1H, d, *J* = 2.1 Hz, H-8), 6.19 (1H, d, *J* = 2.1 Hz, H-6), 5.61 (1H, d, *J* = 7.5 Hz, H-1''), 5.23 (1H, d, *J* = 1.6 Hz, H-1'''), 4.50 (1H, d, *J* = 1.7 Hz, H-1'''') (Fig. 9). ^13^C-NMR (125 MHz, CD_3_OD) *δ*: 161.2 (C-2), 134.3 (C-3), 179.3 (C-4), 163.2 (C-5), 99.8 (C-6), 165.7 (C-7), 94.8 (C-8), 158.5 (C-9), 105.9 (C-10), 123.2 (C-1'), 132.2 (C-2', 6'), 159.0 (C-4'), 116.1 (C-3', 5'), 100.5 (C-1''), 79.9 (C-2''), 78.9 (C-3''), 71.9 (C-4''), 77.1 (C-5''), 68.3 (C-6''), 102.6 (C-1'''), 72.4 (C-2'''), 72.3 (C-3'''), 74.0 (C-4'''), 69.9 (C-5'''), 17.6 (C-6'''), 102.3 (C-1''''), 72.1 (C-2''''), 72.3 (C-3''''), 73.8 (C-4''''), 69.7 (C-5''''), 17.8 (C-6'''') (Fig. 10).

Compound 6 was identified as Kaempferol 3-neohesperidoside 7-glucoside based on ^1^H-NMR, ^13^C-NMR data and references(Wu, Dushenkov, Ho, & Sang, 2009). Kaempferol 3-neohesperidoside 7-glucoside (**6**): yellow amorphous powder, ESI-MS *m/z* 779 [M + Na]^+^, C_33_H_40_O_20_. ^1^H-NMR (500 MHz, DMSO-*d*_6_) *δ*: 12.56 (1H, s, 5-OH), 8.00 (2H, d, *J* = 8.9 Hz, H-2', 6'), 6.84 (2H, d, *J* = 8.9 Hz, H-3', 5'), 6.74 (1H, d, *J* = 2.1 Hz, H-8), 6.38 (1H, d, *J* = 2.1 Hz, H-6), 5.60 (1H, d, *J* = 7.5 Hz, H-1''), 5.08 (1H, d, *J* = 1.6 Hz, H-1'''), 5.03 (1H, b rs, H-1''''), 0.71 (3H, d, *J* = 6.1 Hz, H-CH_3_) (Fig. 11). ^13^C-NMR (125 MHz, DMSO-*d*_6_) *δ*: 156.8 (C-2), 133.1 (C-3), 177.5 (C-4), 160.9 (C-5), 99.4 (C-6), 162.8 (C-7), 94.5 (C-8), 156.8 (C-9), 105.7 (C-10), 120.8 (C-1'), 130.9 (C-2', 6'), 160.1 (C-4'), 115.2 (C-3', 5'), 100.6 (C-1''), 73.1 (C-2''), 76.5 (C-3''), 70.2 (C-4''), 77.5 (C-5''), 60.8 (C-6''), 98.3 (C-1'''), 69.6 (C-2'''), 70.6 (C-3'''), 70.6 (C-4'''), 68.4 (C-5'''), 17.3 (C-6'''), 99.4 (C-1''''), 73.1 (C-2''''), 76.5 (C-3''''), 69.2 (C-4''''), 76.4 (C-5''''), 60.7 (C-6'''') (Fig. 12).

Compound 7 was identified as Isoorientin 2''-*O*-rhamnoside based on ^1^H-NMR, ^13^C-NMR data and references(Prinz, Ringl, Huefner, Pemp, & Kopp, 2007). Isoorientin 2''-*O*-rhamnoside (**7**): yellow amorphous powder, ESI-MS *m/z* 617 [M + Na]^+^, C_27_H_30_O_15_. ^1^H-NMR (500 MHz, DMSO-*d*_6_) *δ*: 7.39 (1H, d, *J* = 8.3, 2.3 Hz, H-6'), 7.37 (1H, d, *J* = 2.3 Hz, H-2'), 6.86 (1H, d, *J* = 8.3 Hz, H-5'), 6.63 (1H, s, H-3), 6.44 (1H, b rs, H-8), 5.01 (1H, s, H-1'''), 4.65 (1H, d, *J* = 9.9 Hz, H-1'') (Fig. 13). ^13^C-NMR (125 MHz, DMSO-*d*_6_) *δ*: 163.4 (C-2), 102.5 (C-3), 181.9 (C-4), 161.3 (C-5), 108.7 (C-6), 163.4 (C-7), 92.9 (C-8), 156.3 (C-9), 103.6 (C-10), 121.0 (C-1'), 112.9 (C-2'), 145.9 (C-3'), 150.2 (C-4'), 116.1 (C-5'), 118.9 (C-6'), 71.6 (C-1''), 74.6 (C-2''), 70.7 (C-3''), 70.4 (C-4''), 81.4 (C-5''), 61.7 (C-6''), 100.4 (C-1'''), 70.2 (C-2'''), 70.9 (C-3'''), 71.6 (C-4'''), 68.3 (C-5'''), 17.6 (C-6''') (Fig. 14).

Compound 8 was identified as Sinensin based on ^1^H-NMR, ^13^C-NMR data and references( Neacsu et al., 2007). Sinensin (**8**): yellow amorphous powder, ESI *m*/*z* 473 [M + Na]^+^, C_21_H_22_O_11_. ^1^H-NMR (500 MHz, CD_3_OD) *δ*: 7.35 (2H, d, *J* = 8.6 Hz, H-2', 6'), 6.82 (2H, d, *J* = 8.9 Hz, H-3', 5'), 6.22 (1H, d, *J* = 2.2 Hz, H-6), 6.19 (1H, d, *J* = 2.2 Hz, H-8), 5.01 (1H, d, *J* = 11.8 Hz, H-2), 4.96 (1H, d, *J* = 7.4 Hz, H-1''), 4.59 (1H, d, *J* = 11.8 Hz, H-3), 3.86 (1H, dd, *J* = 12.2, 2.1 Hz, H-6''a), 3.66 (1H, dd, *J* = 12.2, 5.6 Hz, H-6''b) (Fig. 15). ^13^C-NMR (125 MHz, CD_3_OD) *δ*: 85.1 (C-2), 73.8 (C-3), 199.4 (C-4), 164.8 (C-5), 98.3 (C-6), 167.3 (C-7), 97.0 (C-8), 164.3 (C-9), 103.5 (C-10), 129.1 (C-1'), 130.4 (C-2', 6'), 116.1 (C-3', 5'), 159.3 (C-4'), 101.3 (C-1''), 74.6 (C-2''), 77.8 (C-3''), 71.1 (C-4''), 78.3 (C-5''), 62.3 (C-6'') (Fig. 16).

Compound 9 was identified as Kurarinone based on ^1^H-NMR, ^13^C-NMR data and references(Kang et al., 2000). Kurarinone (**9**): colorless powder, ESI *m*/*z* 461 [M + Na]^+^, C_26_H_30_O_6_. ^1^H-NMR (500 MHz, CD_3_OD) *δ*: 7.39 (1H, d, *J* = 8.4 Hz, H-6'), 6.47 (1H, d, *J* = 2.3 Hz, H-3'), 6.44 (1H, dd, *J* = 8.4, 2.3 Hz, H-5'), 6.20 (1H, s, H-6), 5.60 (1H, dd, *J* = 13.1, 2.7 Hz, H-2), 4.98 (1H, t, *J* = 6.9 Hz, H-4''), 4.58 (1H, br. s, H-9''a), 4.55 (1H, br. s, H-9''b), 3.72 (3H, s, 5-OCH_3_), 2.83 (1H, dd, *J* = 16.1, 13.1 Hz, H-3a), 1.64 (3H, s, H-10''), 1.55 (3H, s, H-7''), 1.47 (3H, s, H-6'') (Fig. 17). ^13^C-NMR (125 MHz, Acetone-*d*_6_) *δ*: 75.0 (C-2), 45.7 (C-3), 189.4 (C-4), 162.4 (C-5), 93.4 (C-6), 163.8 (C-7), 108.5 (C-8), 161.1 (C-9), 106.1 (C-10), 118.3 (C-1'), 155.9 (C-2'), 103.3 (C-3'), 159.1 (C-4'), 107.7 (C-5'), 128.4 (C-6'), 28.0 (C-1''), 47.7 (C-2''), 31.8 (C-3''), 124.5 (C-4''), 131.5 (C-5''), 25.8 (C-6''), 17.8 (C-7''), 149.2 (C-8''), 111.1 (C-9''), 19.1 (C-10''), 55.7 (OCH_3_) (Fig. 18).

Compound 10 was identified as 1-*O*-Vanilloylglucose based on ^1^H-NMR, ^13^C-NMR data and references(Dini, Tenore, & Dini, 2004). 1-*O*-Vanilloylglucose (**10**): white powder, ESI-MS *m*/*z* 353 [M + Na]^+^, C_14_H_18_O_9_. ^1^H-NMR (500 MHz, CD_3_OD) *δ*: 7.64 (1H, dd, *J* = 8.3, 2.0 Hz, H-6), 7.60 (1H, d, *J* = 2.0 Hz, H-2), 6.85 (1H, d, *J* = 8.3 Hz, H-5), 5.68 (1H, d, *J* = 8.0 Hz, H-1'), 3.90 (3H, s, H-OCH_3_) (Fig. 19). ^13^C-NMR (125 MHz, CD_3_OD) *δ*: 121.5 (C-1), 113.8 (C-2), 148.9 (C-3), 153.8 (C-4), 116.1 (C-5), 125.8 (C-6), 166.8 (C-7), 56.4 (C-OCH_3_), 96.1 (C-1'), 74.1 (C-2'), 78.8 (C-3'), 71.1 (C-4'), 78.1 (C-5'), 62.3 (C-6') (Fig. 20).


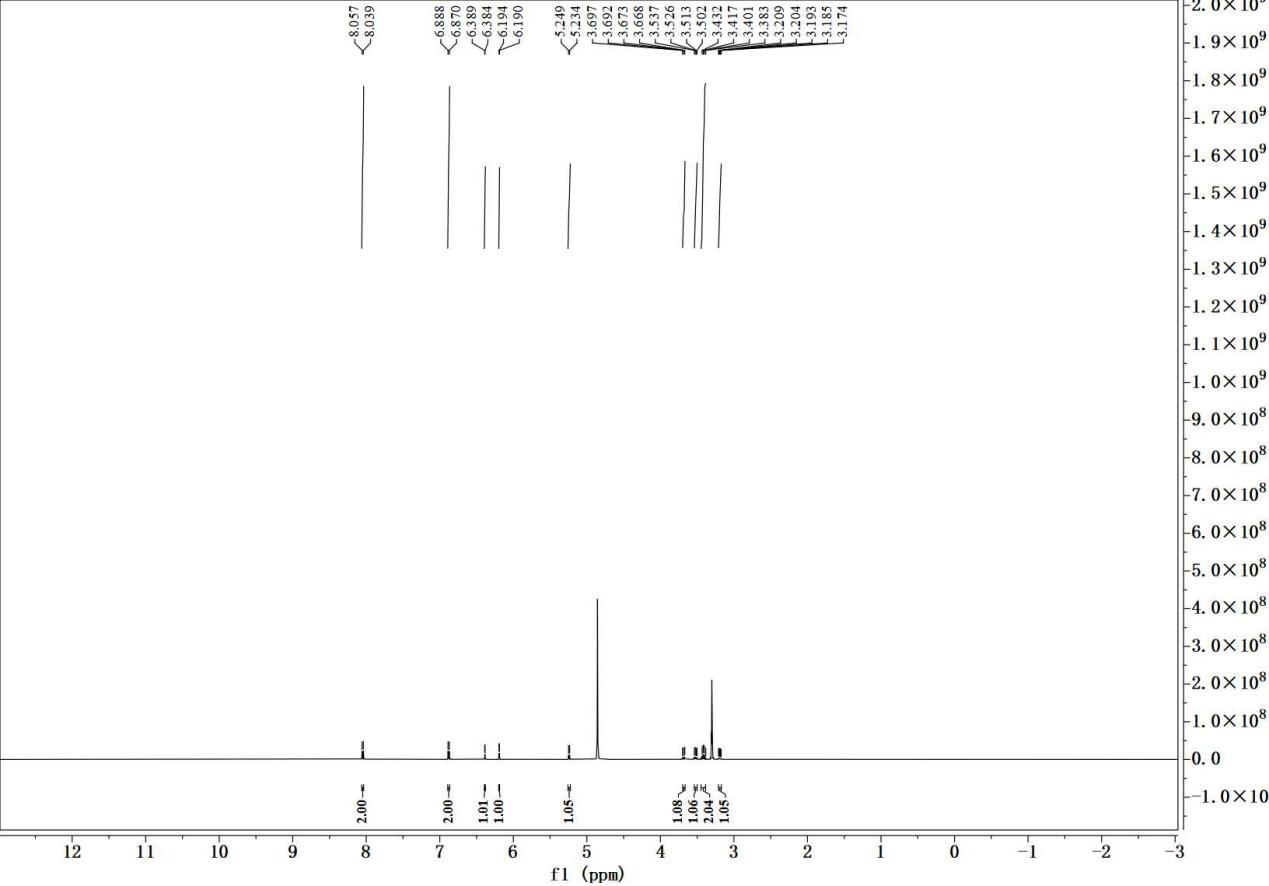


**Fig.1.** ^1^H-NMR (500 MHz, CD_3_OD) Spectrum of Compound 1
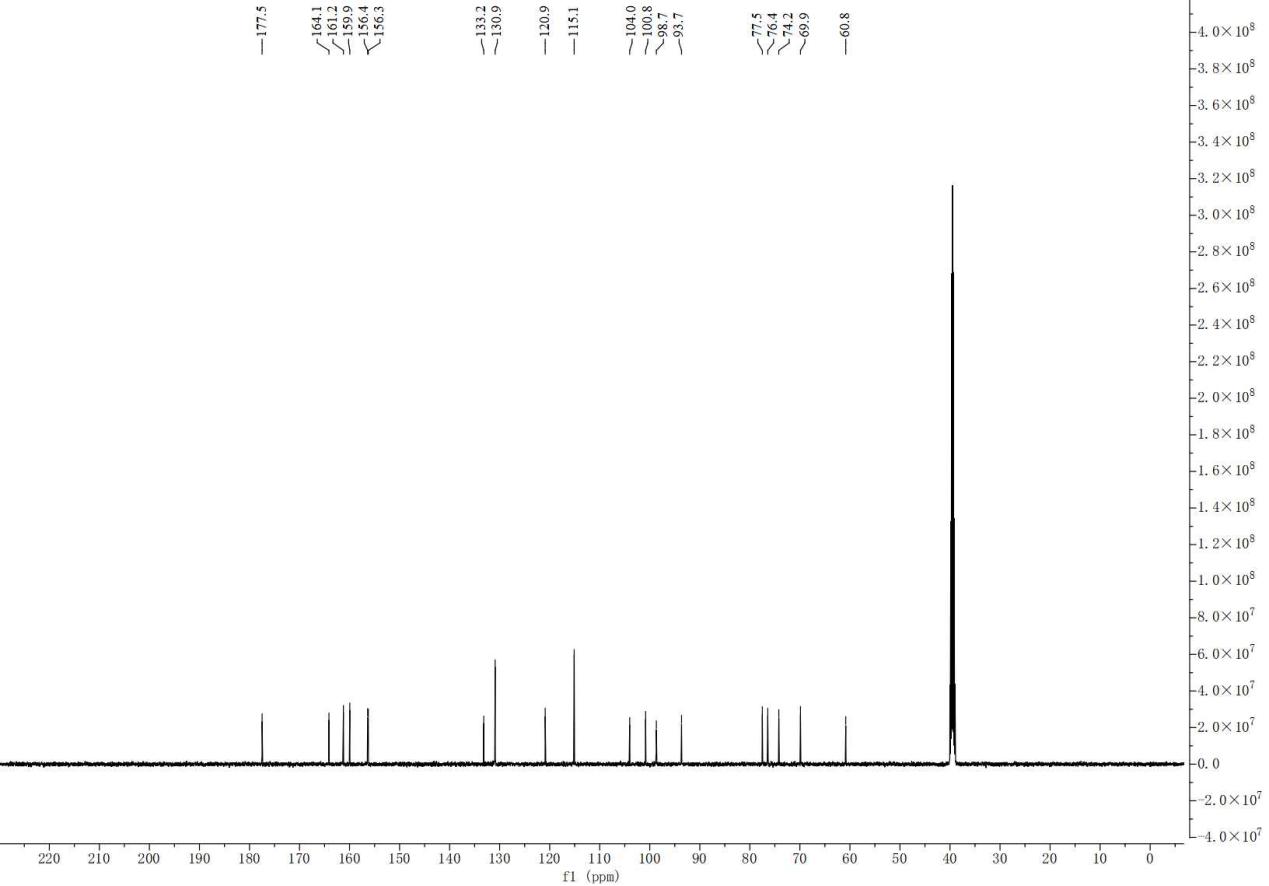


**Fig.2.** ^13^C-NMR (125 MHz, DMSO-*d*_6_) Spectrum of Compound 1


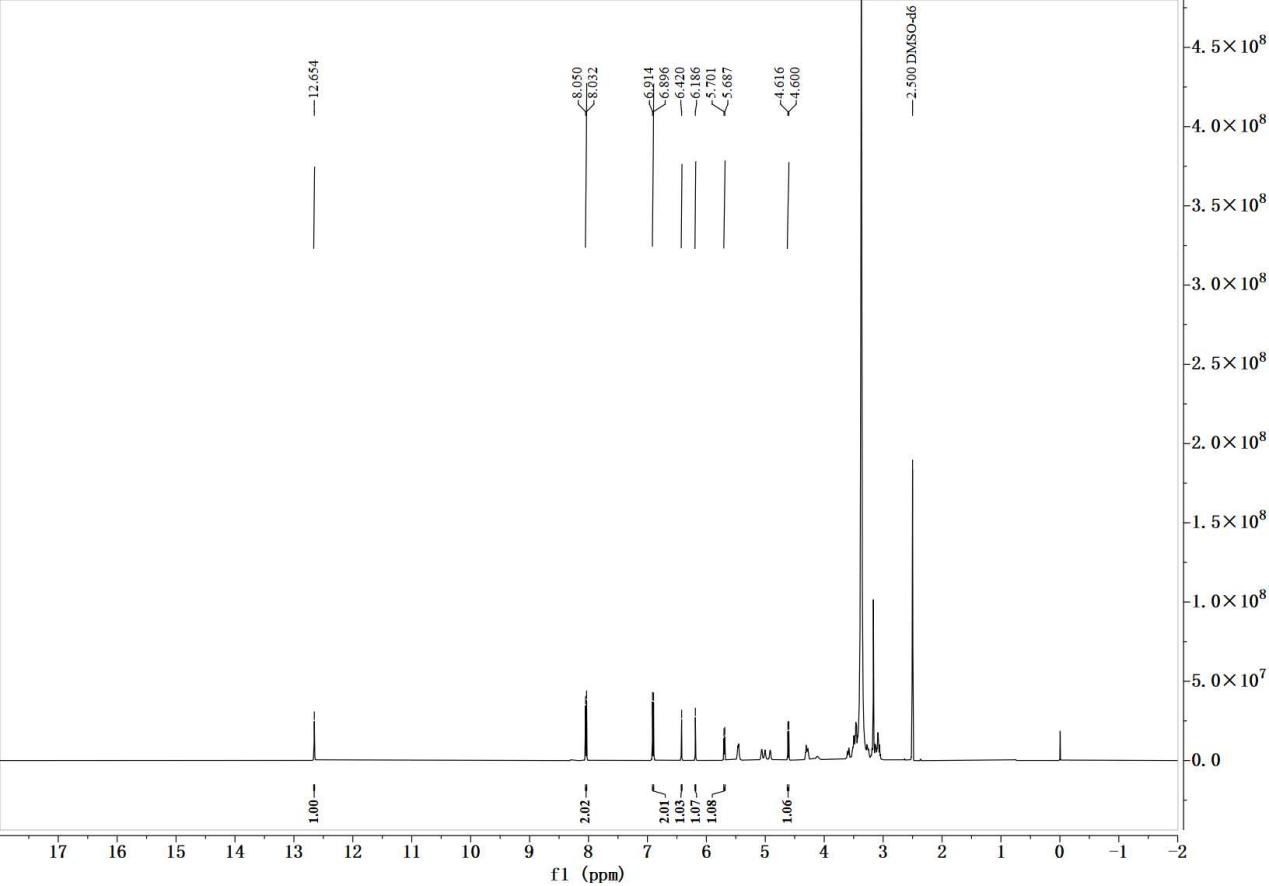


**Fig.3.** ^1^H-NMR (500 MHz, DMSO-*d*_6_) Spectrum of Compound 2

**
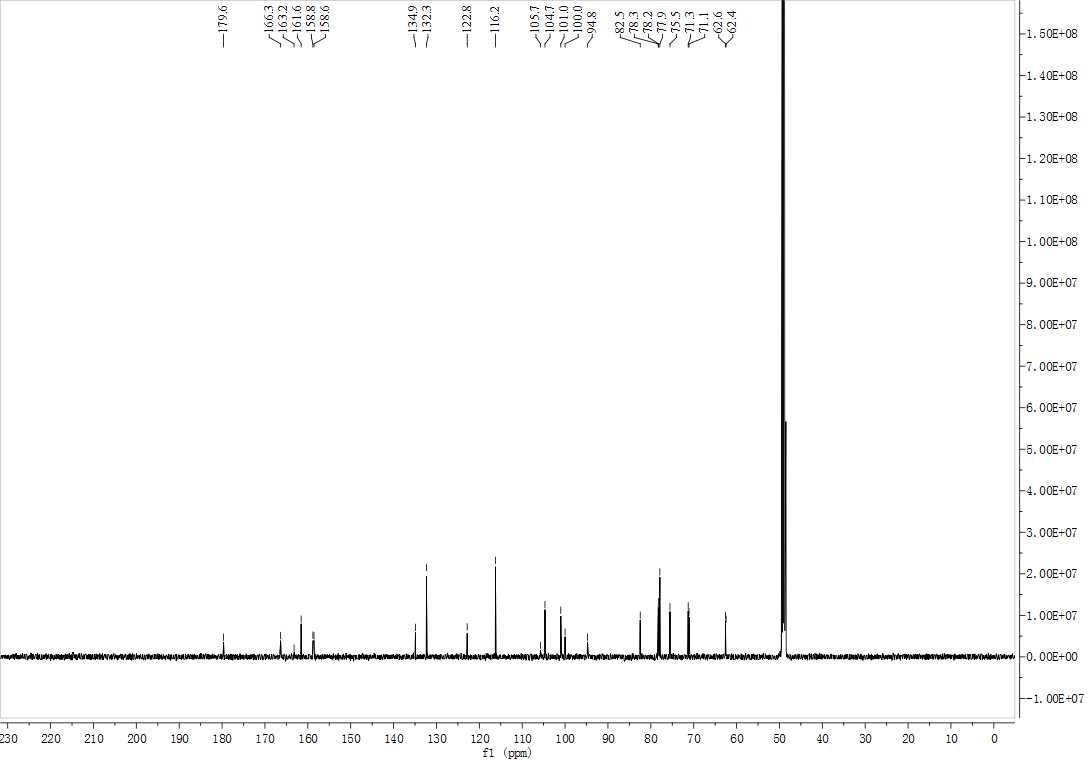
**

**Fig.4.** ^13^C-NMR (125 MHz, DMSO-*d*_6_) Spectrum of Compound 2


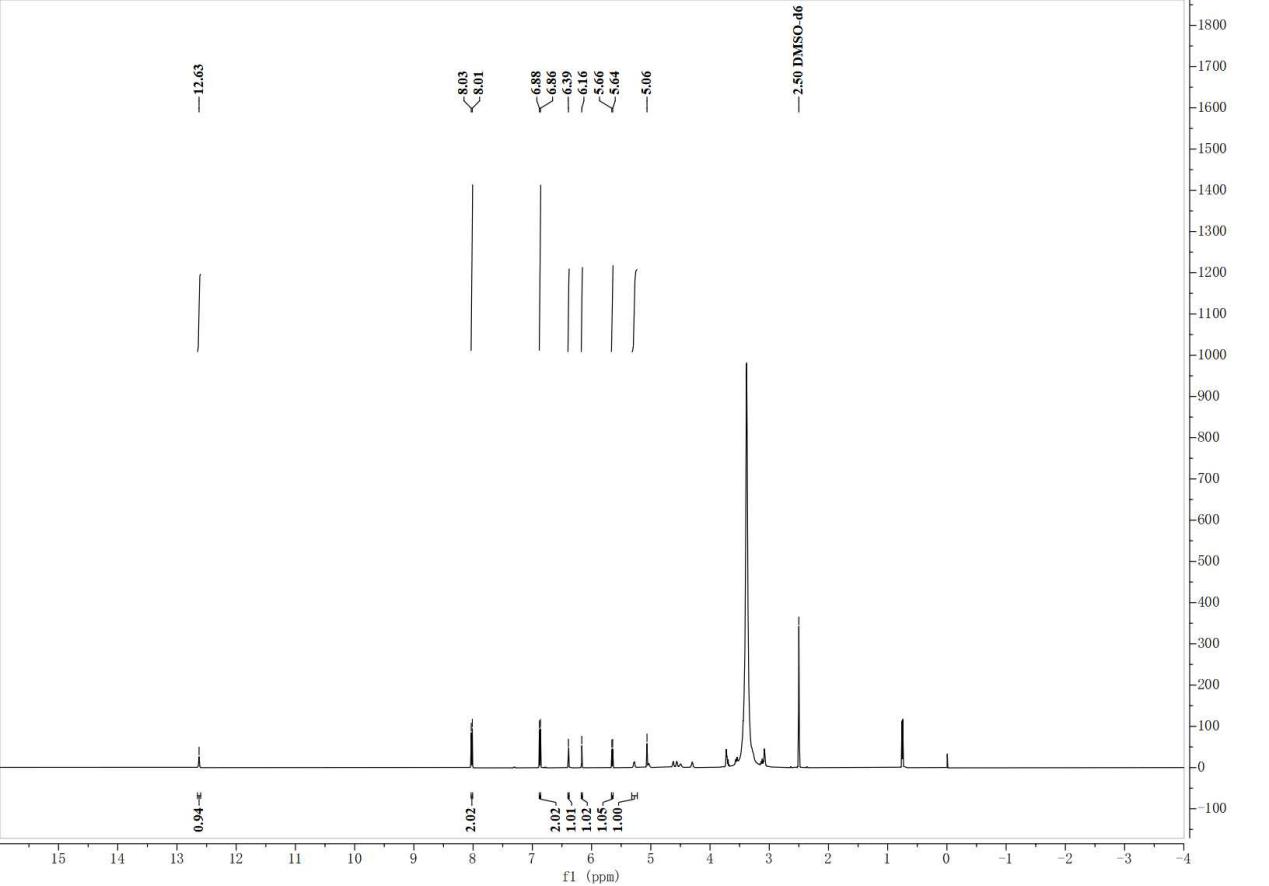


**Fig.5.** ^1^H-NMR (500 MHz, DMSO-*d*_6_) Spectrum of Compound 3

**
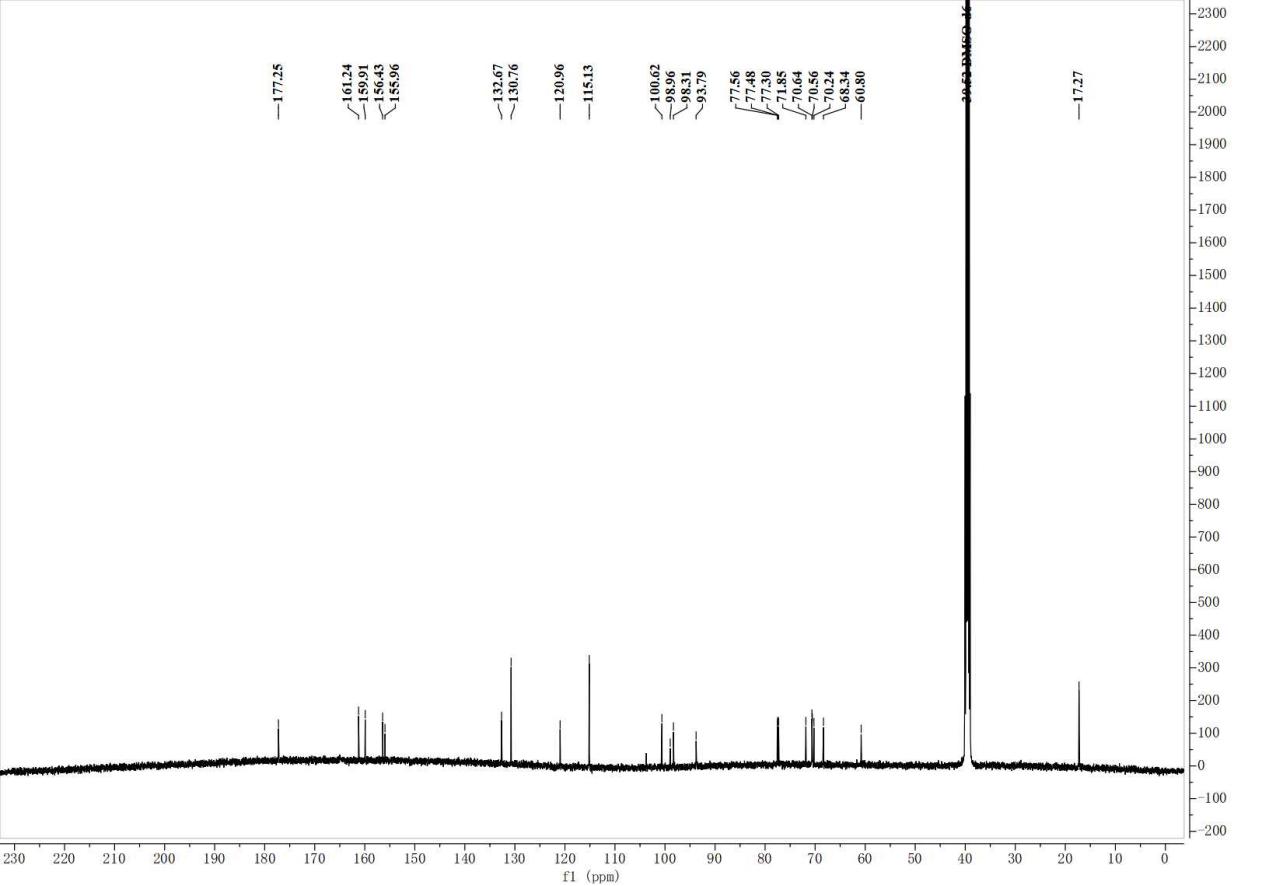
**

**Fig.6.** ^13^C-NMR (125 MHz, DMSO-*d*_6_) Spectrum of Compound 3

**
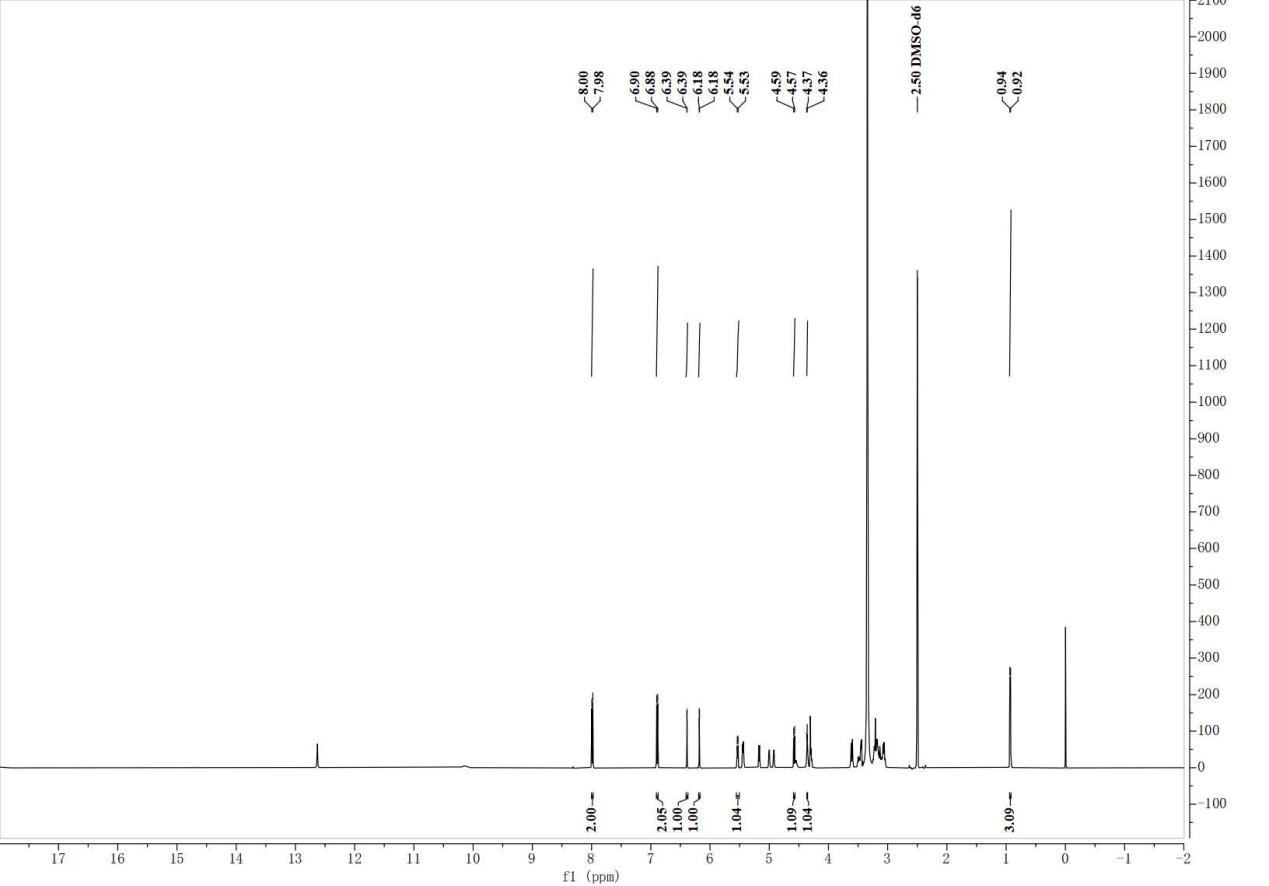
**

**Fig.7.** ^1^H-NMR (500 MHz, DMSO-*d*_6_) Spectrum of Compound 4

**
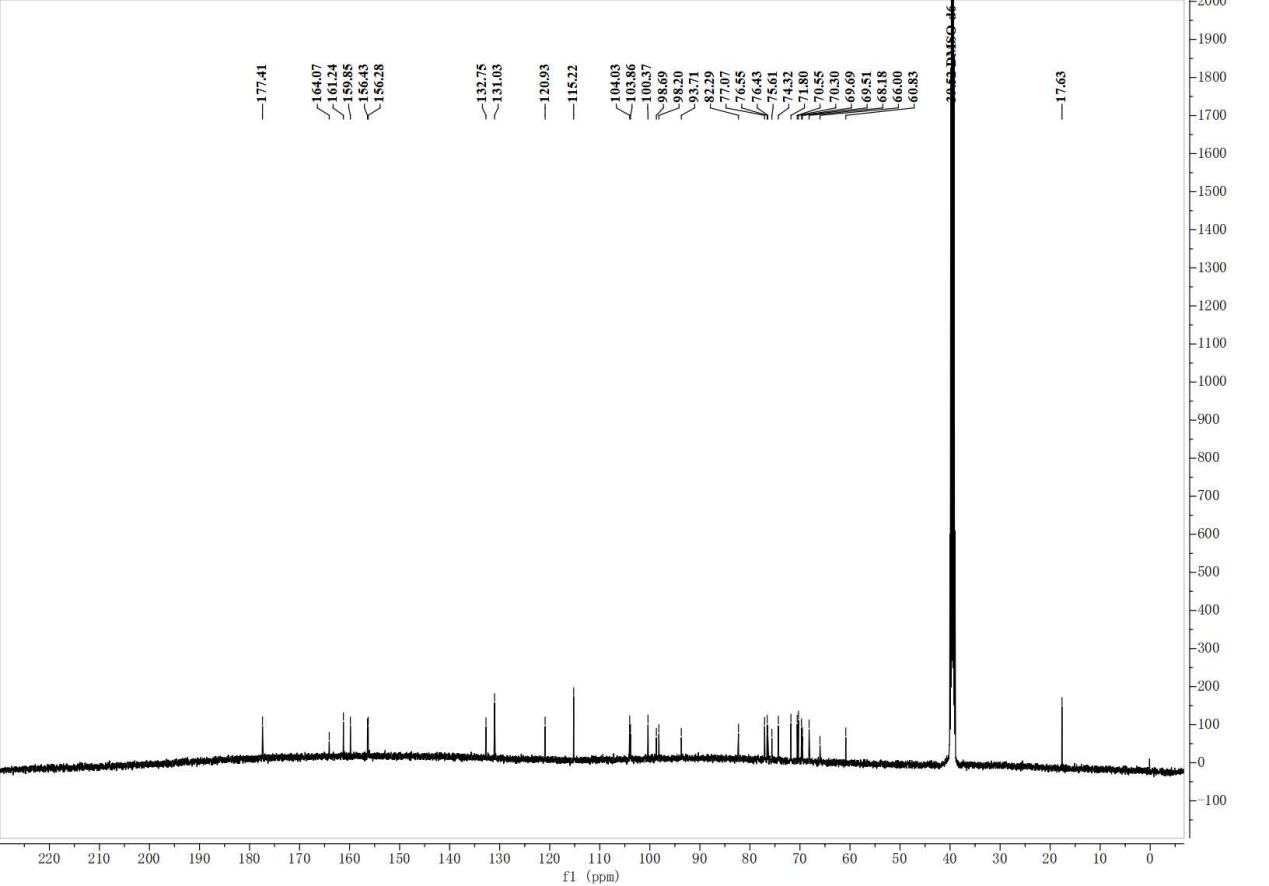
**

**Fig.8.** ^13^C-NMR (125 MHz, DMSO-*d*_6_) Spectrum of Compound 4


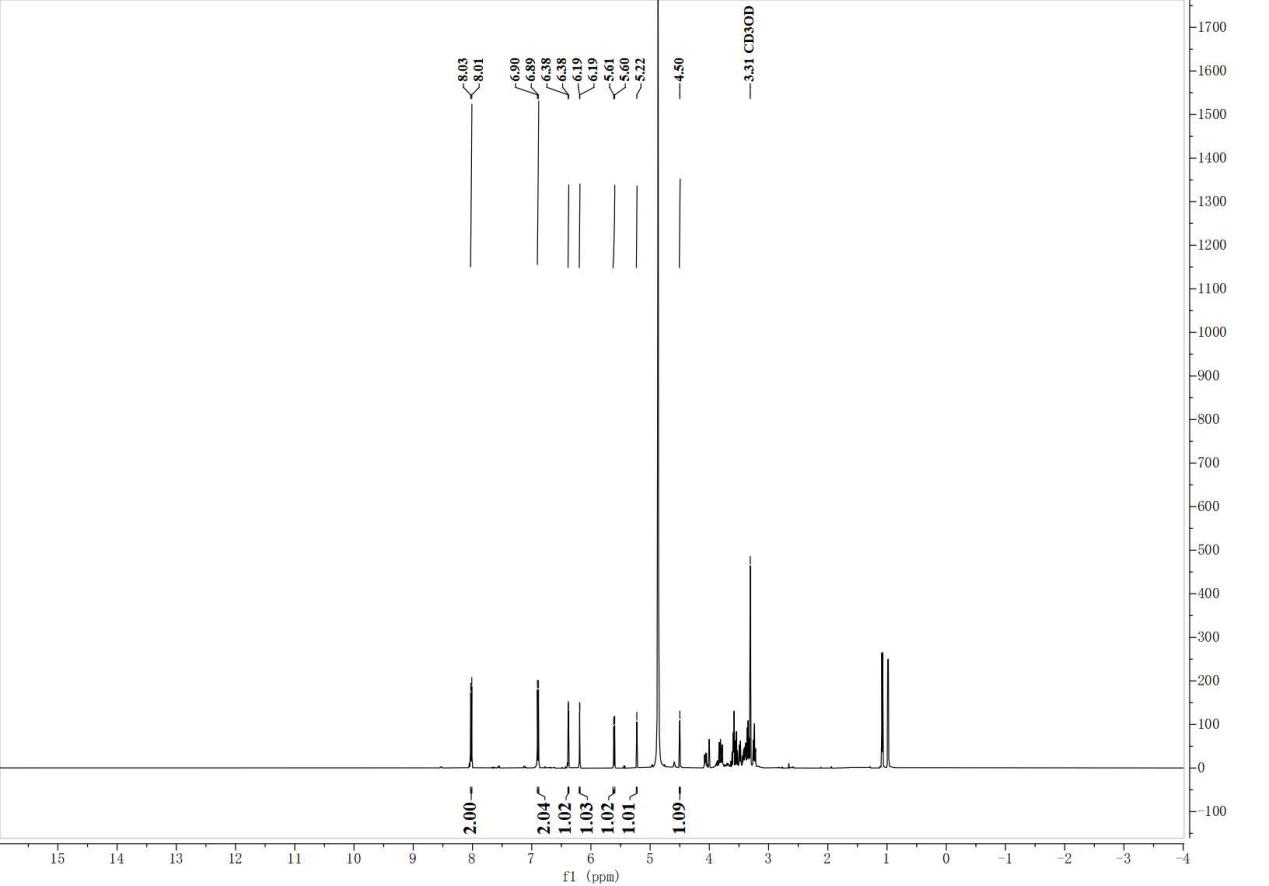


**Fig.9.** ^1^H-NMR (500 MHz, CD_3_OD) Spectrum of Compound 5

**
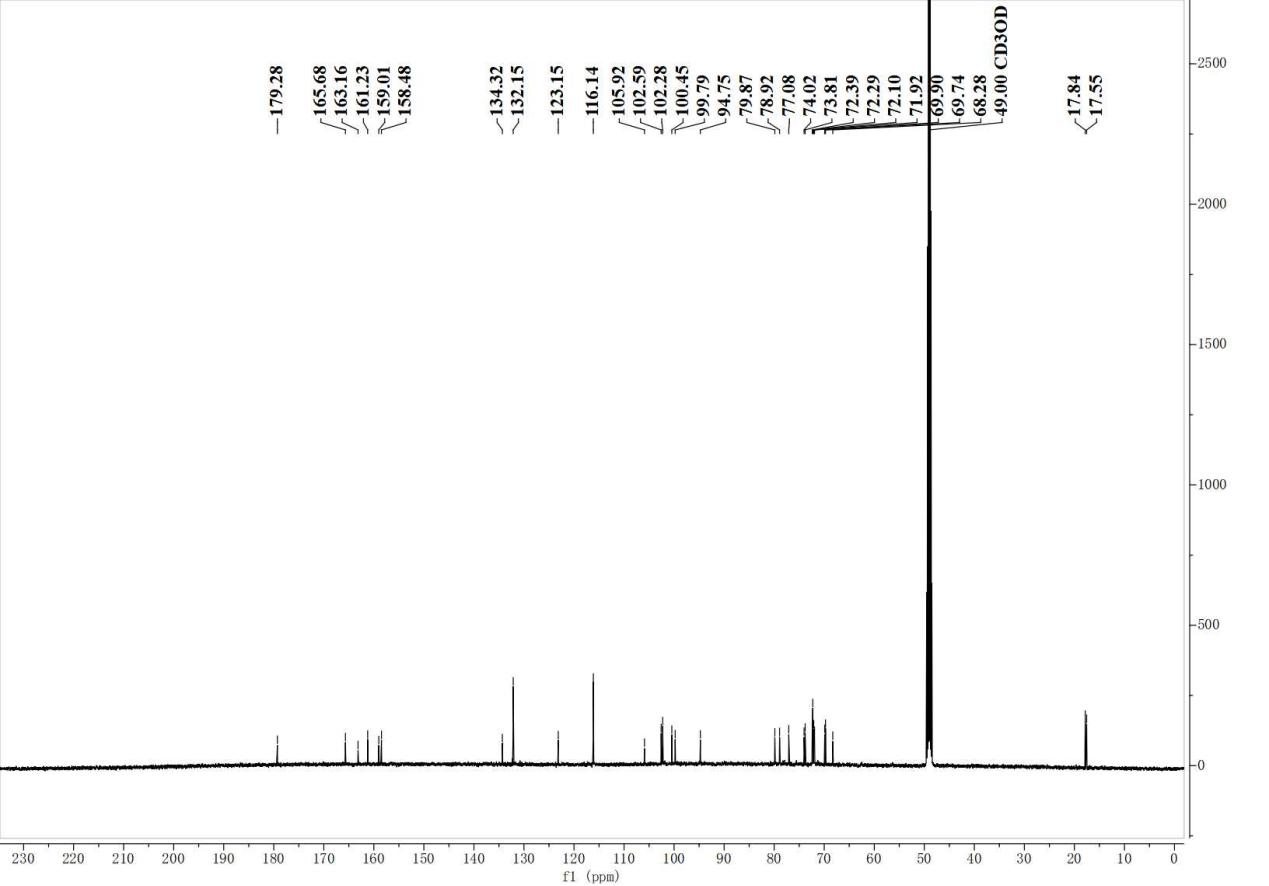
**

**Fig.10.** ^13^C-NMR (125 MHz, CD_3_OD) Spectrum of Compound 5

**
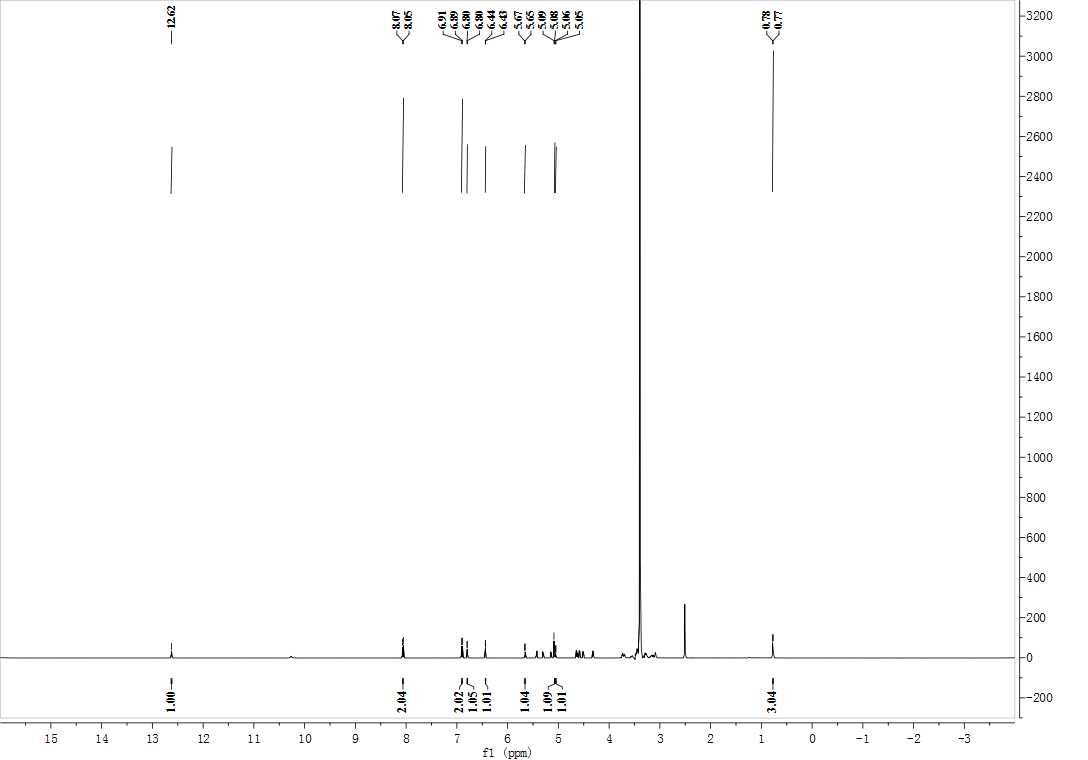
**

**Fig.11.** ^1^H-NMR (500 MHz, DMSO-*d*_6_) Spectrum of Compound 6

**
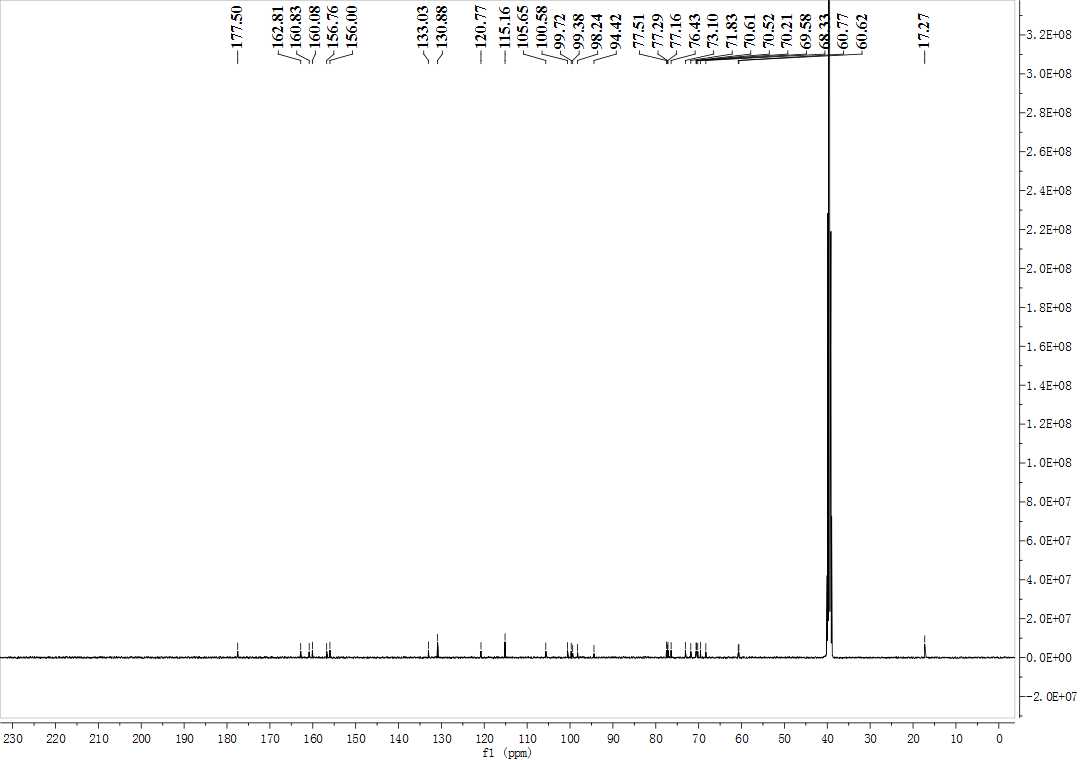
**

**Fig.12.** ^13^C-NMR (125 MHz, DMSO-*d*_6_) Spectrum of Compound 6


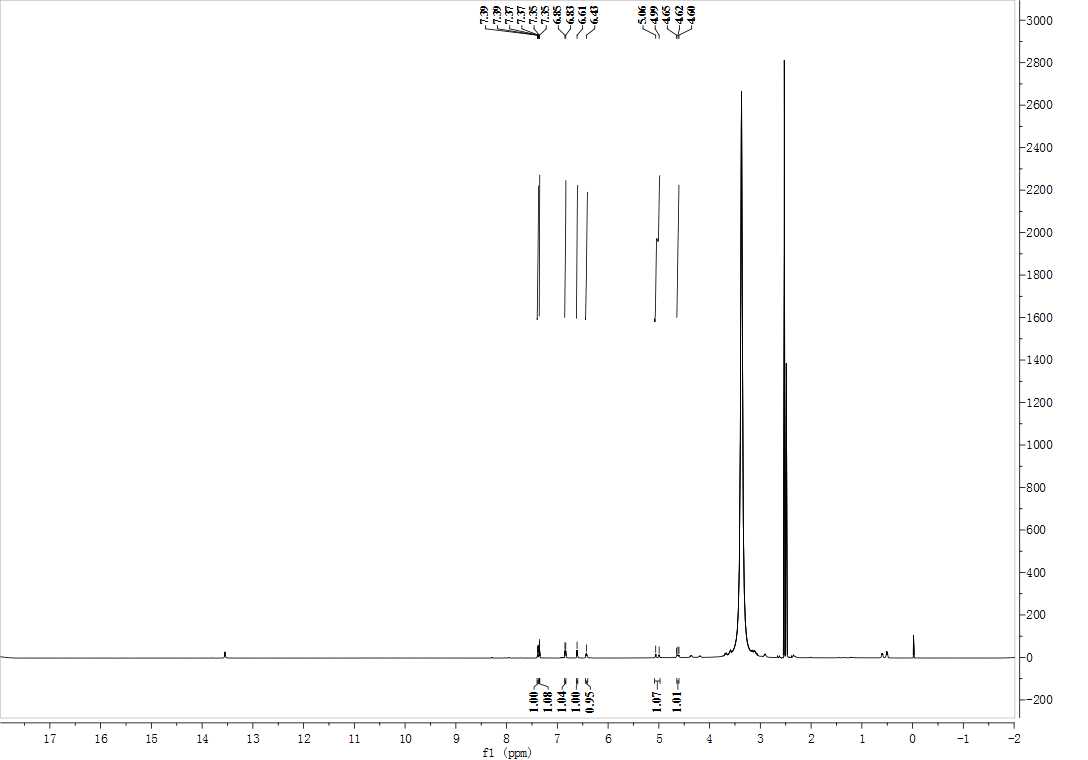


**Fig.13.** ^1^H-NMR (500 MHz, DMSO-*d*_6_) Spectrum of Compound 7

**
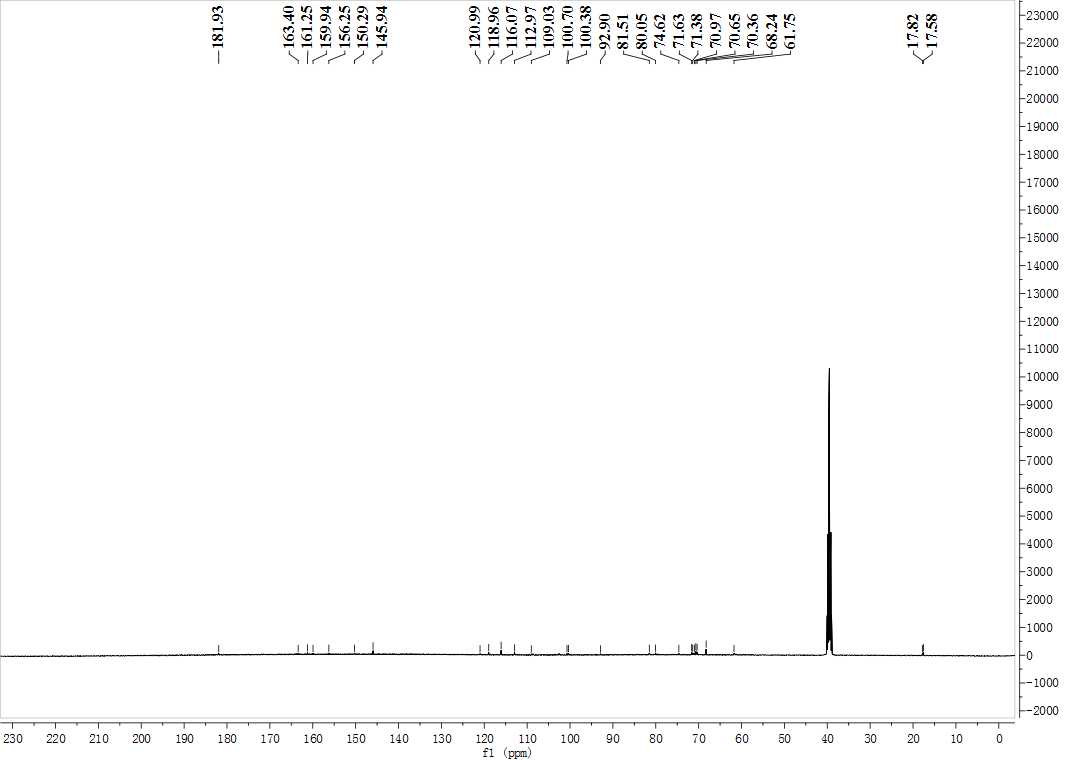
**

**Fig.14.** ^13^C-NMR (125 MHz, DMSO-*d*_6_) Spectrum of Compound 7


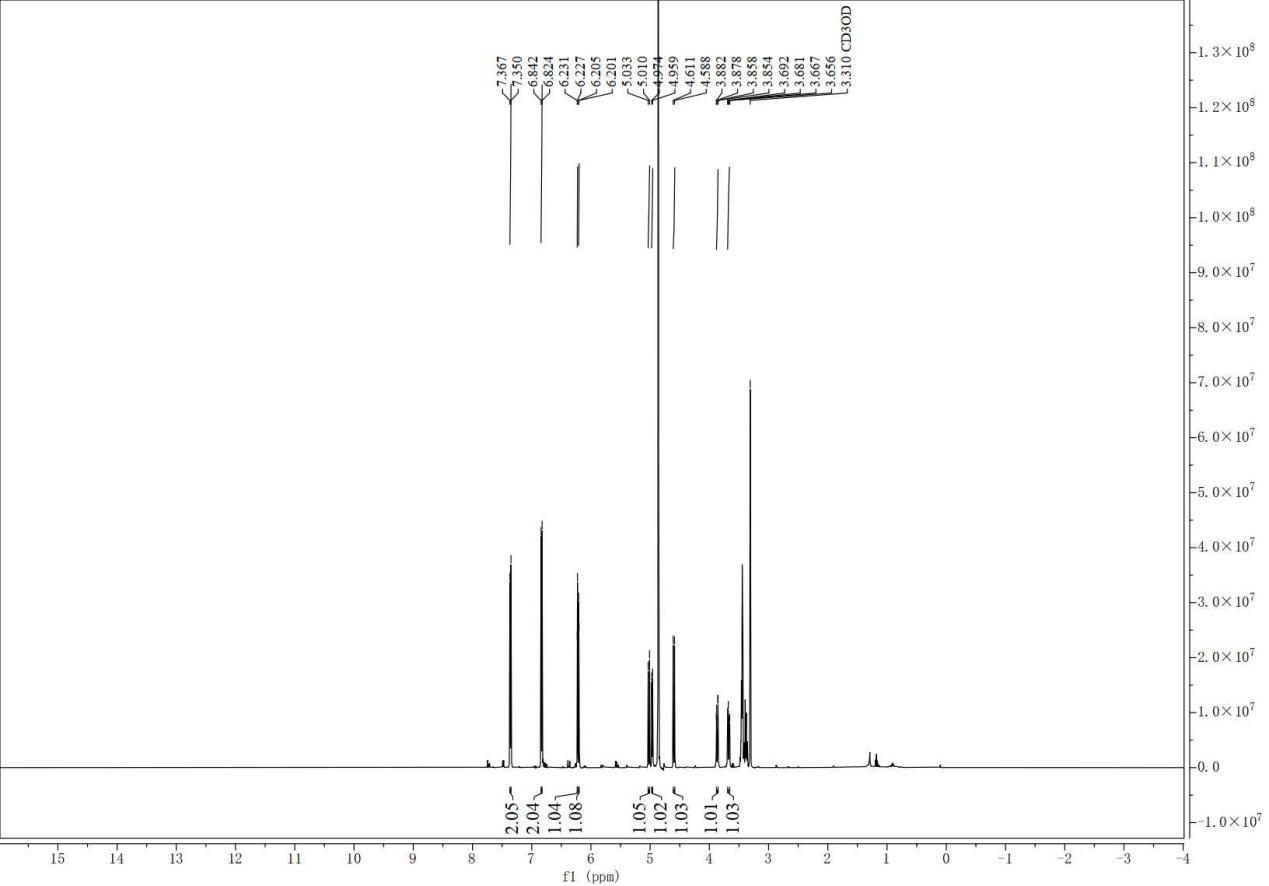


**Fig.15.** ^1^H-NMR (500 MHz, CD_3_OD) Spectrum of Compound 8

**
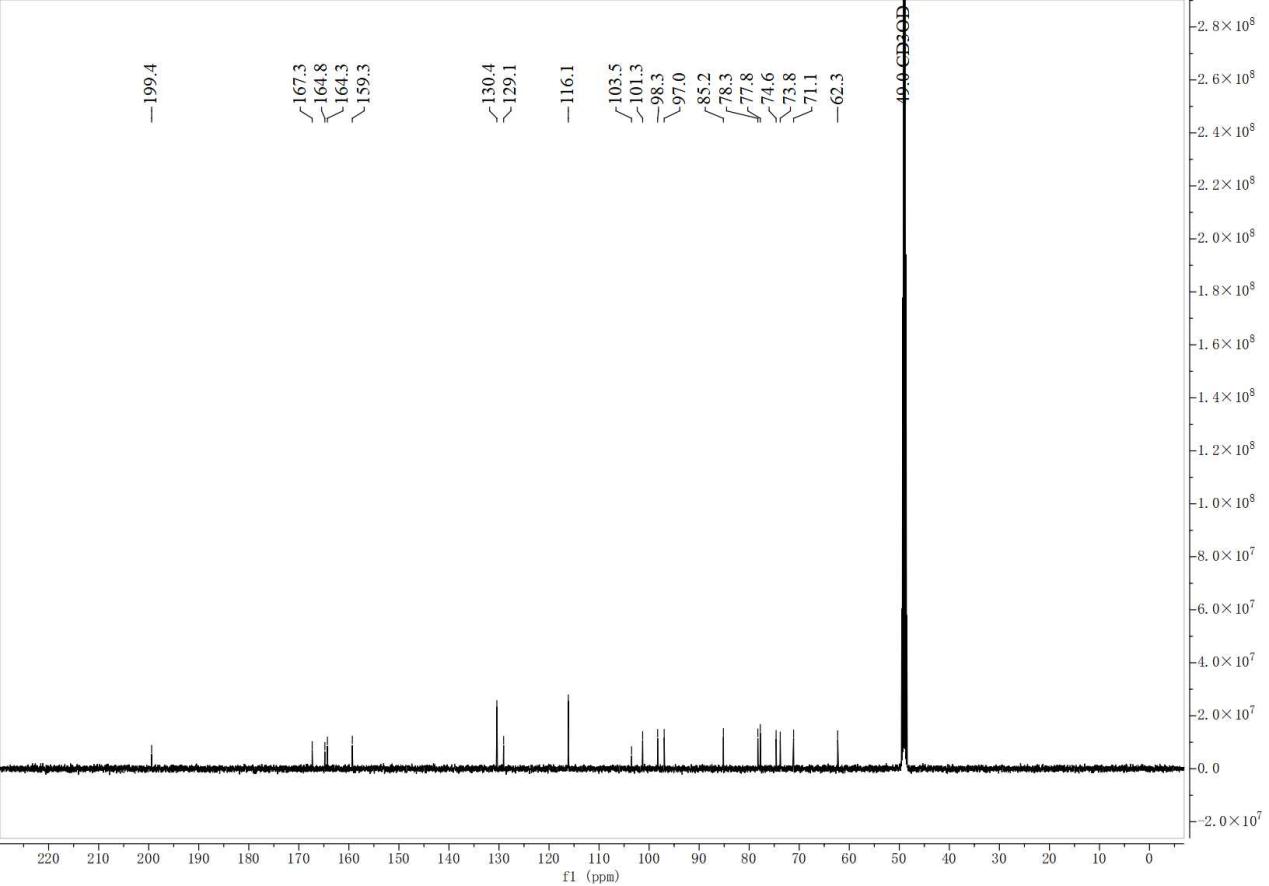
**

**Fig.16.** ^13^C-NMR (125 MHz, CD_3_OD) Spectrum of Compound 8


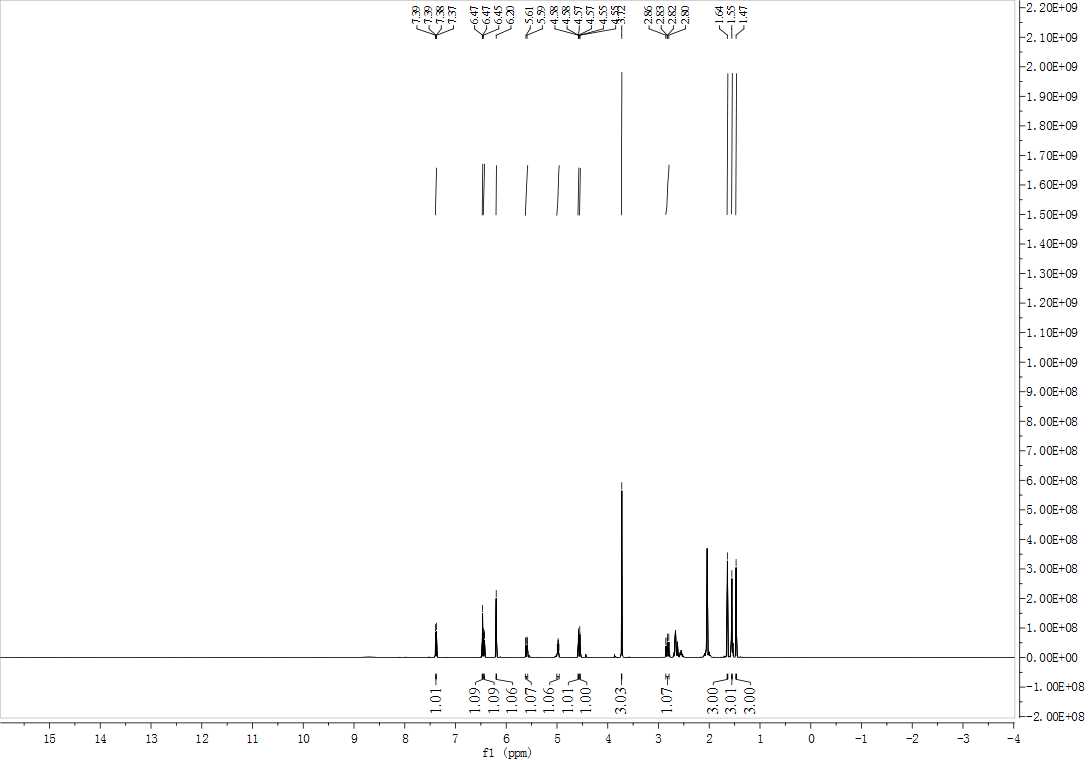


**Fig.17.** ^1^H-NMR (500 MHz, CD_3_OD) Spectrum of Compound 9

**
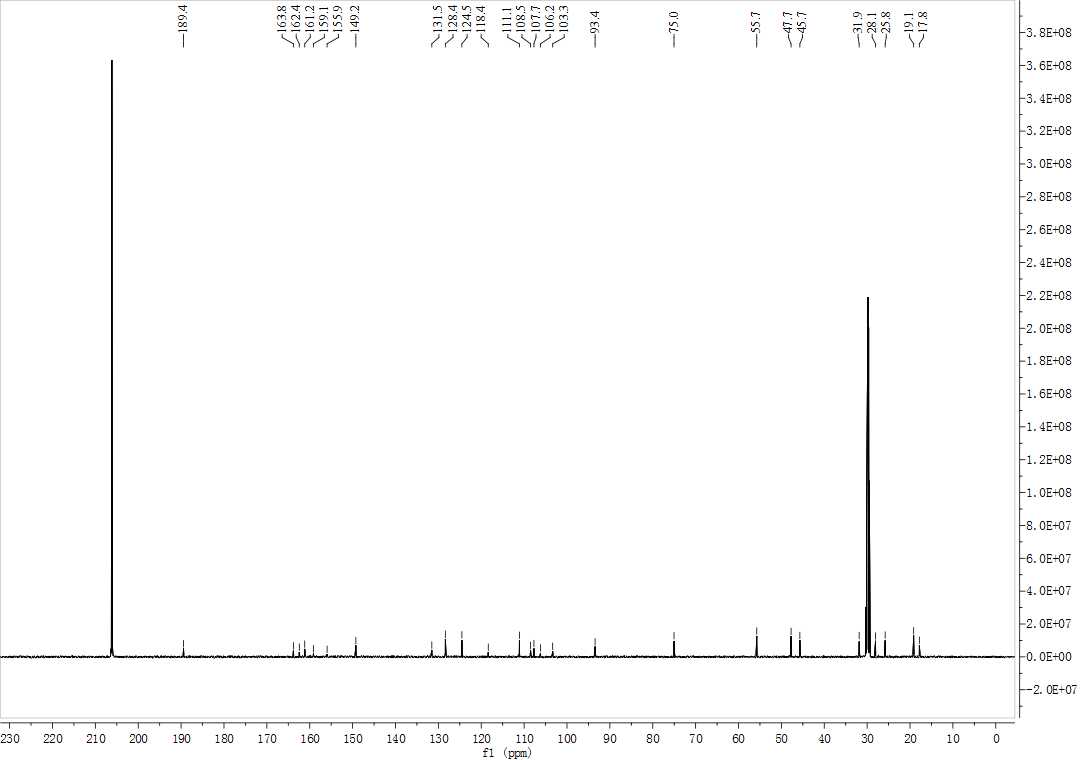
**

**Fig.18.** ^13^C-NMR (125 MHz, Acetone-*d*_6_) Spectrum of Compound 9


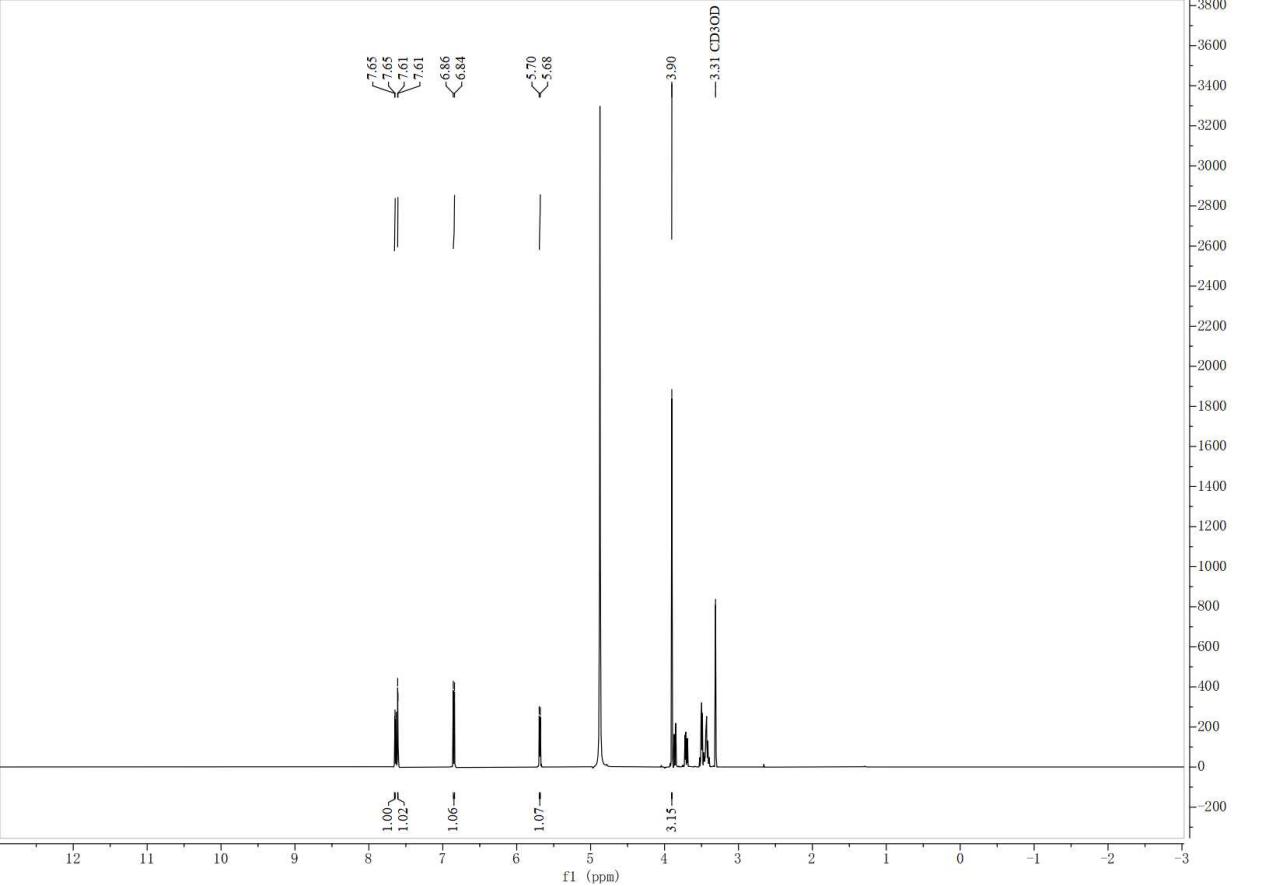


**Fig.19.** ^1^H-NMR (500 MHz, CD_3_OD) Spectrum of Compound 10

**
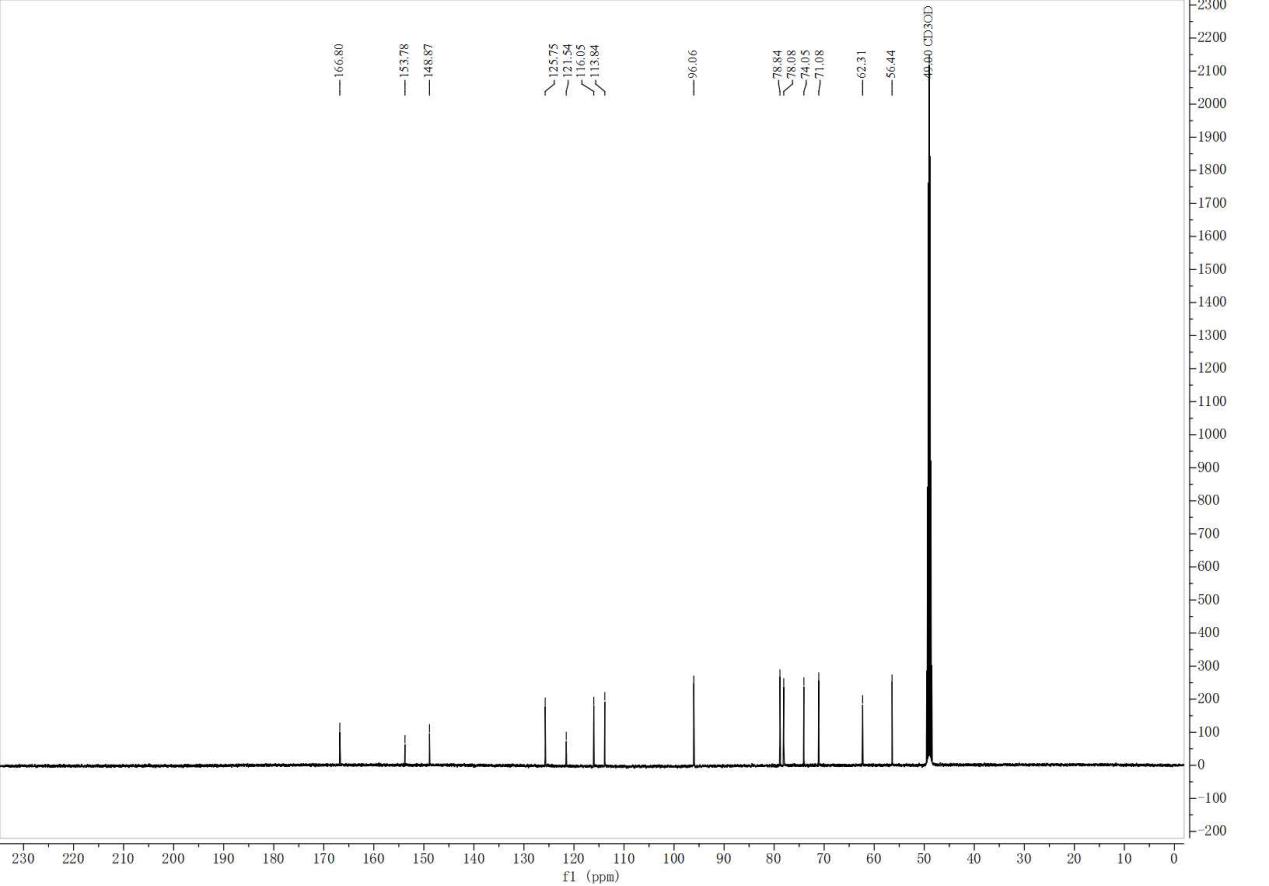
**

**Fig.20.** ^13^C-NMR (125 MHz, CD_3_OD) Spectrum of Compound 10

**References**

Budzianowski J. (1990). Kaempferol glycosides from Hosta ventricosa. Phytochemistry, 29(11), 3643–3647. <https://doi.org/10.1016/0031-9422(90)85292-n>

Dini, I., Tenore, G. C., & Dini, A. (2004). Phenolic constituents of kancolla seeds. Food Chemistry, 84(2), 163-168. <https://doi.org/10.1016/S0308-8146(03)00185-7>

Kang, T. H., Jeong, S. J., Ko, W. G., Kim, N. Y., Lee, B. H., Inagaki, M., et al. (2000). Cytotoxic lavandulyl flavanones from *Sophora flavescens*. Journal of natural products, 63(5), 680–681. <https://doi.org/10.1021/np990567x>

Kazuma, K., Noda, N., & Suzuki, M. (2003). Malonylated flavonol glycosides from the petals of *Clitoria ternatea*. Phytochemistry, 62(2), 229–237. https://doi.org/10.1016/s0031-9422 (02)00486-7

Nørbaek, R., & Kondo, T. (1999). Flavonol glycosides from flowers of *Crocus speciosus* and C. *antalyensis*. Phytochemistry, 51(8), 1113–1119. https://doi.org/10.1016/s0031-9422(99) 00109-0

Neacsu, M., Eklund, P. C., Sjöholm, R. E., Pietarinen, S. P., Ahotupa, M. O., Holmbom, B. R., et al. (2007). Antioxidant flavonoids from knotwood of Jack pine and European aspen. Holz Als Roh Und Werkstoff. <https://doi.org/10.1007/s00107-006-0121-0>

Okuyama, T., Hosoyama K., Hiraga Y., Kurono G., &Takemoto, T. (1978). The constituents of Osmunda spp. II. A new flavonol glycoside of Osmunda asiatica. Chemical & pharmaceutical bulletin, 26(10), 3071-3074. <https://doi.org/10.1248/cpb.26.3071>

Prinz, S., Ringl, A., Huefner, A., Pemp, E., & Kopp, B. (2007). 4'''-Acetylvitexin-2''-*O*-rhamnoside, isoorientin, orientin, and 8-methoxykaempferol-3-*O*-glucoside as markers for the differentiation of *Crataegus monogyna* and *Crataegus pentagyna* from Crataegus laevigata (Rosaceae). Chemistry & biodiversity, 4(12), 2920–2931. https://doi.org/10.1002/cbdv. 200790241

Wolfram, K., Schmidt, J., Wray, V., Milkowski, C., Schliemann, W., & Strack, D. (2010). Profiling of phenylpropanoids in transgenic low-sinapine oilseed rape (*Brassica napus*). Phytochemistry, 71(10), 1076–1084. <https://doi.org/10.1016/j.phytochem.2010.04.007>

Wu, H., Dushenkov, S., Ho, C. T., & Sang, S. (2009). Novel acetylated flavonoid glycosides from the leaves of allium ursinum. Food Chemistry, 115(2), 592-595.https://doi.org/10.1016/ j.foodchem.2008.12.058
